# Assessing health disparities faced by female paid domestic workers in Peru before, during, and after the COVID-19 pandemic

**DOI:** 10.1186/s12939-025-02617-w

**Published:** 2025-11-26

**Authors:** David Vera-Tudela, Maria Kathia Cárdenas, Ramón Díaz, Christopher Meaney, María Lazo-Porras, Viviana Cruzado, Archna Gupta, Janeth Tenorio-Mucha

**Affiliations:** 1 Universidad Peruana Cayetano Heredia , Lima, Peru; 2 CRONICAS Center of Excellence in Chronic Diseases, Universidad Peruana Cayetano Heredia, Lima, Peru; 3 Department of Family and Community Medicine, University of Toronto, Toronto, Canada; 4 Pontificia Universidad Católica del Perú, Lima, Peru; 5 Upstream Lab, MAP Centre of Urban Health Solutions, Li Ka Shing Knowledge Institute, St. Michael’s Hospital, Toronto, Canada

**Keywords:** Domestic workers, Women’s health, Health inequalities, Health services accessibility, COVID-19, Social determinants of health, Peru

## Abstract

**Background:**

Female paid domestic workers are among the most vulnerable occupational groups globally, often lacking formal social protection and limited labour rights. The COVID-19 pandemic may have exacerbated these vulnerabilities, yet quantitative evidence from low and middle income countries is scarce. This study examines health disparities in Peru between female paid domestic workers and females employed in the formal service sector before, during, and after the pandemic.

**Methods:**

We used pooled cross sectional data from the Peruvian National Household Survey (ENAHO, 2018–2023). The primary outcomes were self reported illness symptoms and healthcare seeking behaviour. We compared female paid domestic workers to female formal workers in other service occupations–including both personal and nonpersonal services–across three time periods: prepandemic (January 2018 - February 2020), pandemic (March 2020 - October 2022), and postpandemic (November 2022 - December 2023). Analyses involved comparing differences in proportions and conducting Wald tests. We also stratified results by key social determinants of health, including education, ethnicity, age, income, chronic disease status, household head status, and access to labour rights.

**Results:**

Female paid domestic workers reported more illness symptoms and sought less healthcare than females working in nonpersonal service roles, especially during the pandemic. The difference in proportions - 5.9 percentage points (pp.) for illness symptoms and 16.5 pp. for healthcare-seeking behaviour- became smaller after one year. There were no significant differences when comparing female paid domestic workers to other personal service workers. Stratified results indicated that outcome differences between female paid domestic workers and female working in non-personal services were wider among household heads, those with chronic conditions, and those with limited access to labour rights. Post-pandemic disparities were especially pronounced among younger females, low-wage earners, and those with less education.

**Conclusion:**

In Peru, female paid domestic workers experienced persistent health disadvantages before, during, and after the COVID-19 pandemic when compared with females with formal employment. Addressing these disparities requires comprehensive policies that promote formalization and social security coverage to advance progress on Sustainable Development Goal 3.

**Supplementary Information:**

The online version contains supplementary material available at 10.1186/s12939-025-02617-w.

## Background

Globally, approximately 75.6 million people work in domestic labour, of whom seven out of ten are female [1]. Most of these workers, particularly in low- and middle-income countries (LMICs), lack adequate coverage of social protection policies, leaving them without unemployment insurance, access to a retirement pension, paid annual leave, social security, healthcare insurance, and other labour rights.

This study focuses specifically on *female paid domestic workers*, who are defined as females engaged in tasks such as cleaning, cooking, and caregiving in or for a household within an employment relationship and receive monetary compensation. Domestic workers face elevated risks to both mental and physical health, including anxiety, depression, musculoskeletal pain, allergic reactions to cleaning chemicals, and workplace accidents [2–4]. In Latin America, one in four domestic workers reports chronic conditions—such as hypertension, diabetes, or obesity [5].

The COVID-19 pandemic intensified these vulnerabilities, resulting in disproportionate job losses, wage reductions, increased exposure to the virus, and a heightened incidence of abuse and violence against them across Latin America [5–8]. Paid domestic workers, the majority of whom are female, constitute one of the occupational groups most severely affected by the COVID-19 pandemic, primarily due to their limited access to social protection compared to other female workers [5, 9].

Peru was among the countries most affected by the COVID-19 pandemic in terms of health and economic impacts ( [10]– [11]). National survey data revealed that over 40% of female domestic workers lost their jobs during the first year of the pandemic [12], similar to domestic workers in India (45%) [4]. Those who remained often accepted live-in arrangements, leading to longer hours and increased exposure to exploitative conditions [13]– [14]. For live-out domestic workers, the pandemic disrupted their transportation and mobility patterns, negatively affecting their family responsibilities, household budgets [15], and higher exposure to burnout and stress [16].

This study investigates changes in health disparities in Peru between 2018 and 2023, comparing self-reported illness symptoms and healthcare-seeking behaviour among female paid domestic workers and female workers formally employed in service occupations. We propose that domestic workers were more negatively affected by the pandemic compared to females with formal employment, due to differences in social security and labour rights. Using six years of national data and the Social Determinants of Health framework [20], we examine health outcomes across pre-, mid- and post- pandemic periods, based on structural and intermediary factors.

By focusing on these two occupational groups, the study addresses a critical evidence gap on how the COVID-19 pandemic reinforced existing patterns of health disparities. This analysis aligns with the World Health Organization’s concerns about the increased vulnerabilities of low-income essential workers [19] and it responds to calls for stronger monitoring of health inequalities under Sustainable Development Goal 3. Although focused on the Peruvian context, our findings provide insights with broader relevance for other LMICs facing similar challenges in protecting informally employed workers.

## Methods

### Study design

This research uses pooled cross-sectional data from 2018 to 2023 to examine the changes in disparities on illness symptoms and healthcare-seeking behaviour across three periods (before, during, and after the COVID-19 pandemic) between female paid domestic workers (hereafter, “domestic workers”) and female workers formally employed in non-domestic occupations within the service sector (hereafter, “formal workers”).

We calculate differences in proportions to estimate the disparities on the outcome variables between domestic workers and formal workers per period. Additionally, we calculate stratified differences by key individual characteristics based on the conceptual framework of the Social Determinants of Health [20] to examine health inequalities between domestic workers and formal workers in non-domestic occupations. This framework distinguishes between contextual, structural, and intermediary determinants of health, highlighting how social, economic, and political conditions shape unequal exposures and vulnerabilities across population groups [18]. This approach allows us to explore how intersecting social determinants influence health outcomes within this population and enhances the analysis of intra-group inequalities from an intersectional perspective.

### Setting

For this study, the pandemic period is defined from March 2020 to October 2022, when Peru was under a state of National Emergency due to COVID-19, with nationwide restrictions on mobility, services, and economic activity. The pre-pandemic period includes January 2018 to February 2020, and the post-pandemic period spans from November 2022 to December 2023.

### Data source

The data comes from the National Household Survey (ENAHO, by its Spanish acronym) administered annually in Peru by the National Institute of Statistics and Informatics. The survey’s target population includes private households and all their regular inhabitants at the national level. The sample size is approximately 34,500 households surveyed annually from 2018 to 2023. The sampling design employed a three-stage stratified cluster sampling methodology, ensuring that the indicators are representative at both national level and within specific geographic domains [19].

### Participants

The ENAHO enables the identification of household members who self-reported domestic work as their primary occupation during the week preceding the interview. The criteria we used to include participants in our study were: (i) being female, (ii) living in urban areas (settlements with 2,000 inhabitants or more) at the time of the interview, and (iii) being occupied. Additionally, due the design of the occupational question in the survey, only individuals aged 14 years or above were included.

Domestic workers who did not report working hours or any labour income in their main occupation and those who had another primary occupation but offered domestic activities as a side job were excluded. When household variables were used for difference stratification, the analysis excluded female paid domestic workers who live or spend the night in the household of their employer (live-in domestic workers). This is due to the fact that the household variables collected in the survey pertain to the employer'shousehold, rather than that of the domestic worker.

Formal workers include females engaged in any other service activity (excluding domestic work). Following the definition used by the Ministry of Labour in Peru, formal employment definition includes those with one of the following criteria: (i) employees with employer-paid social security contributions, or (ii) self-employed workers registered with the National Tax Authority in Peru. The same inclusion criteria used for domestic workers are applied to formal workers.

To explore heterogeneity in occupational characteristics and socio-economic positioning, we classified formal workers in two comparison groups based on the International Standard Industrial Classification of All Economic Activities (ISIC), Revision 4:

- Comparison Group 1 (CG1): female workers with formal employment in personal services activities, excluding domestic work, which typically involve similar occupations to domestic workers (e.g. beauty services, laundry, restaurants).
- Comparison Group 2 (CG2): female workers with formal employment in non-personal services activities, which tend to involve professional occupations (e.g. education institutions, healthcare providers, financial companies).

In total, CG1 encompasses four categories from personal services and six from non-personal services. Supplementary File 1 includes more details about the composition of the comparison groups.

### Outcomes

Health outcomes were measured via binary indicators of health status and access to healthcare services, following the comprehensive national health equity surveillance framework suggested by the World Health Organization [18]:

- Illness symptoms: was positive for participants who self-reported symptoms, illness, or relapse of a chronic condition in the four weeks before the interview.
- Healthcare-seeking behaviour: was positive for participants who sought care from a healthcare facility (public or private) for any symptoms or illness in the four weeks preceding the interview.

Both outcome variables were measured for domestic workers and both comparison groups across periods and by individual characteristics. Supplementary File 2 provides additional details about the construction of the outcome variables based on the ENAHO’s questionnaire questions.

### Variables

According to each component of the Social Determinants on Health framework [20], the following proxy variables are used for measurement and included inthe stratification analysis:

- Element 1: Socioeconomic and political context. This analysis aims to identify disparities in outcomes for both domestic workers and formal workers residing in Lima - the capital of Peru - and to compare these findings with those from other urban areas in the country. Lima serves as the seat of government institutions, accommodates nearly30% of Peru’s population and accounts for about 36% of the nation’s gross domestic product [21]. Consequently, residents benefit from enhanced labour market prospects, broader access to healthcare services, and more comprehensive basic services compared to individuals living in other cities across Peru.
- Element 2: Structural determinants and socioeconomic position. We utilize three proxy variables to reflect the socioeconomic position of domestic workers in Peru, as these are associated with their access to material resources and opportunities for improved health and well-being. The selected variables include: (i) Self-identified ethnic origin (Indigenous or Afro-descendant vs. white/mixed/others), (ii) Highest education attaintment (incomplete basic education vs. complete basic education or higher), (iii) Quintiles of hourly wage (first and second quintiles vs. fourth and fifth quintiles).
- Element 3: Intermediary determinants. In accordance with the SDH framework, the analysis addresses four variables: (i) age group (< 34, 35–49, ≥ 50 years) as a demographic factor; (ii) presence of chronic condition as abiological factor; (iii) head of household status; and (iv) number of labour rights fulfilled out of a possible five (two or fewer vs. three or more) as psychosocial stressors, which represent income generation responsibility and income insecurity. The five labour rights under Peruvian labour regulations identified from available data are: possession of a labour contract; affiliation with a retirement scheme; employer-provided social security; receipt of an additional salary for National Holiday allowance; and receipt of an additional salary for Christmas allowance.

Supplementary File 2 provides additional details regarding the definition of the quantitative variables based on the ENAHO’s questionnaire questions.

### Statistical methods

First, we calculate the proportions of (i) illness symptoms, and (ii) health-seeking behaviour for each level of the employment group (i.e. domestic and formal workers) and by study time period (i.e. pre-pandemic, pandemic, post-pandemic). We present point estimates of these proportions (with 95% confidence intervals) for each group and time period, and assess differences using tests for differences in proportions (Wald tests) as well as graphical comparisons. 

To conduct the Wald tests, we ran logistic regressions separately for each period, using each binary outcome variable as the dependent variable and including dummy variables for the comparison groups. The Wald tests were then applied independently to compare the coefficient for domestic work and with those of each comparison group, assessing whether they are statistically different. A non-significant result would indicate no meaningful differences in the outcome variable between groups. The null hypotheses for each period are as follows:

$$\:{H}_{o}^{}:\left\{\begin{array}{c}{{Z}}_{DW}^{p1}-{{Z}}_{FW}^{p1}=0\\\:{{Z}}_{DW}^{p2}-{{Z}}_{FW}^{p2}=0\\\:{{Z}}_{DW}^{p3}-{{Z}}_{FW}^{p3}=0\\\:\end{array}\right\}$$

where $\:{Z}^{pi}$ is a vector including the proportion of the outcome variables in the period $\:i$ (p1 for pre-pandemic, p2 for pandemic, and p3 for post-pandemic) for each group (FW for formal workers and DW for domestic workers).

Finally, we calculated proportions stratified by the variables used as proxies for each component of the Social Determinants in Health Framework described in the previous section, with 95% confidence intervals reported. All calculations and estimates accounted for the complex survey design of ENAHO, including stratification, clustering, and population weights. Data analysis was performed using Stata (version 18) and figures in R (version 4.4.3).

## Results

Domestic workers make up 5.8% of the female workforce in urban Peru and domestic work constitutes the fourth most important activity for females working in the service sector and the second most important among personal services activities after “Accommodation and food services activities”. Among the non-personal services, the most frequent activities are “Financial and insurance, real estate, professional, scientific, and administrative support activities” and “Education”. Females working in non-personal services have the highest rate of formal employment (60.9%), followed by domestic workers (13.3%) and female workers in non-domestic personal services (11.0%). Supplementary File 3 shows the distribution of females working within the service sector by activity and period.

Across the six-year sample, a total of 4,957 domestic workers, 2,109 females with formal employment in non-domestic personal services (comparison group 1) and 17,556 females with formal employment in non-personal services met the inclusion criteria (comparison group 2). All three groups have similar distribution between periods (40% in pre-pandemic, 40% in pandemic and 20% in post-pandemic roughly).

### Characteristics of study population

Table 1 presents the primary characteristics of the sample of domestic workers and the comparison groups for all periods. Based on six years of data, domestic workers have a higher proportion of younger (14–19 years old) and older females (50 years or more) compared with both comparison groups. Similarly, a higher percentage of domestic workers live in Lima (Peru’s capital) and other urban areas along the country’s coast than formal workers. More than 26% of domestic workers self-identify as Indigenous (e.g., Quechua, Aymara, or Amazonian ethnic groups) or as an Afro-descendant, compared with 23% in comparison group 1 and 18% in comparison group 2.

**Table 1:** Characteristics of study population for all periods (% females of each group – weighted sample)

Characteristic	Domestic workers	CG1 - non-domestic personal services	CG2 - non-personal services
Sample observations (weighted)	1,958,852	667,897	5,378,878
Sample observations (non-weighted)	4,957	2,109	17,556
**Age group**			
14–19	6.33	3.4	0.52
20–34	23.32	37.66	37.04
35–49	38.55	36.68	37.35
50+	31.8	22.26	25.09
**Geographic domain**			
Coast	24.29	20.77	19.54
Highlands	12.25	20.41	20.5
Amazon	7.62	8.95	7.2
Lima city (capital)	55.84	49.87	52.76
**Educational attainment**			
Complete primary or less	24.68	10.23	1.26
Incomplete secondary	17.09	8.7	1.55
Complete secondary	38.88	35.64	11.33
Higher education	19.35	45.43	85.86
**Self-reported ethnic group**			
Indigenous	20.44	18.42	15.69
Afrodescendant	6.09	4.76	3.16
White and others	15.99	12.86	9.9
Mixed background	57.48	63.96	71.25
**Hourly wage average by quintile** (US$)			
1Q (lower income)	0.26	0.22	0.24
2Q	0.84	0.84	0.91
3Q	1.40	1.39	1.39
4Q	2.08	2.12	2.15
5Q (higher income)	4.11	6.68	5.60
**Number of labour rights met (out of 5)**			
0	64.81	2.08	0.2
1	16.57	44.32	4.64
2	11.11	16.88	6.83
3	4.35	11.95	15.43
4	2.17	9.5	14.26
5	0.99	15.27	58.64
**Chronic disease condition**			
No	46.47	48.54	44.81
Yes	53.53	51.46	55.19
**Household head** 1/			
No	63.54	69.06	73.99
Yes	36.46	30.94	26.01

1/ For live-out Domestic Workers only. Data source: ENAHO 2018–2023

The most significant gap between domestic workers and the comparison groups are educational attainment and labour rights access. More than 44% of domestic workers did not complete secondary school, compared with 18% of comparison group 1 and less than 3% of comparison group 2. On the other hand, almost 65% of domestic workers cannot access any of the five labour rights measured, compared to 2% for comparison group 1 and less than 1% for comparison group 2. The hourly average wage of domestic workers is close to both comparison groups from the first to the third quantile of the wage’s distribution but is 38% and 27% lower than comparison group 1 and comparison group 2, respectively, for the fifth quintile.

Finally, more than half of domestic workers self-reported a chronic condition, similar to comparison group 2 and slightly higher than comparison group 1. More than 36% of domestic workers are heads of their own households, higher than comparison group 1 (30.9%) and comparison group 2 (26.0%).

To compare the sample over time, data were pooled according to the corresponding period. Aside from the number of observations, the sample composition across periods is quite similar. Comparing the pandemic period with the pre-pandemic period, the most notable differences across all groups include lower hourly wages, a higher percentage of females self-reporting a chronic condition, and a greater proportion of females as household heads. In the post-pandemic period, these differences widen compared with the pandemic period. Supplementary File 4 includes the characteristics of the study population by period.

### Illness symptoms

**Fig. 1:**
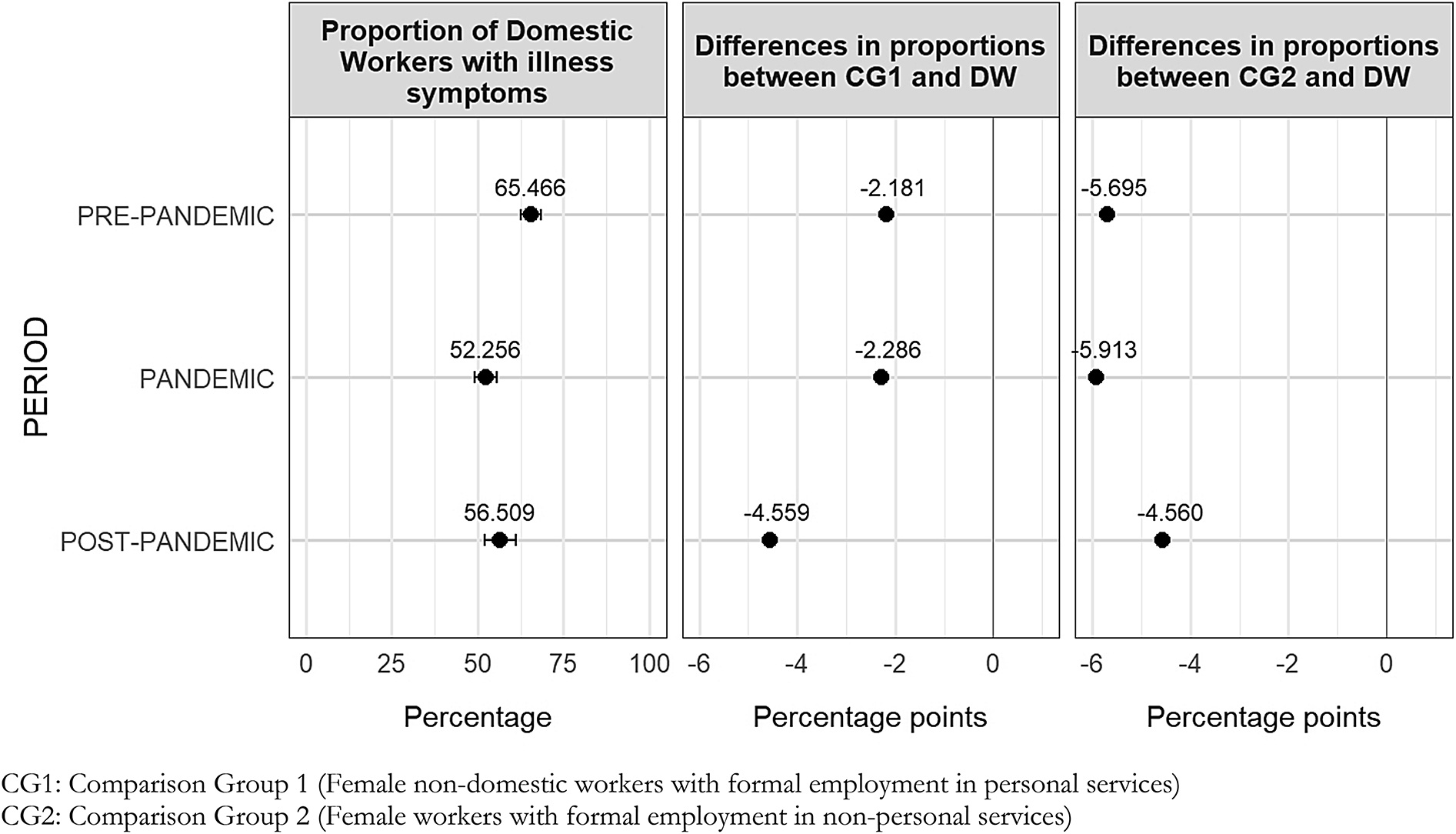
Differences on illness symptoms between female paid domestic workers and comparison groups by period *(proportions presented on percentage scale) *

Figure 1 shows the proportions differences in illness symptoms between domestic workers and comparison groups across each period. At baseline (pre-pandemic period), the percentage of domestic workers with illness symptoms was 65.5% [95% CI: 62.5% − 68.4%], which was statistically similar to comparison group 1 [95% CI: 58.9% − 67.6%] but higher than comparison group 2 [95% CI: 57.9% − 61.6%]. The gap between domestic workers and comparison group 2 is 5.7 pp. which is statistically significant at 95% confidence.

After the pre-pandemic period, the proportion of females with illness symptoms decreased for all groups. During the pandemic period, 52.3% [95% CI: 49.0% − 55.5%] of domestic workers self-reported any illness symptom, and no statistical difference was found with comparison group 1 [95% CI: 43.4% − 56.5%]. The proportion of females incomparison group 2 who reported illness symptoms [95% CI: 44.2% − 48.4%] is significantly lower than that of domestic workers, and the gap was 5.9pp. which was similar to the pre-pandemic period.

In the post-pandemic period, the proportion of females with illness symptoms were higher than in the pandemic period but still lower than the pre-pandemic levels for all groups. Over half of domestic workers (56.5%, [95% CI: 52.0% − 61.0%]) self-reported any illness symptoms, similar to comparison group 1 [95% CI: 44.9% − 59.5%], but significantly higher than comparison group 2 [95% CI: 49.3% − 54.6%]. The gap between domestic workers and comparison group 2 was 4.6 pp., which is smaller than in previous periods.

**Fig. 2:**
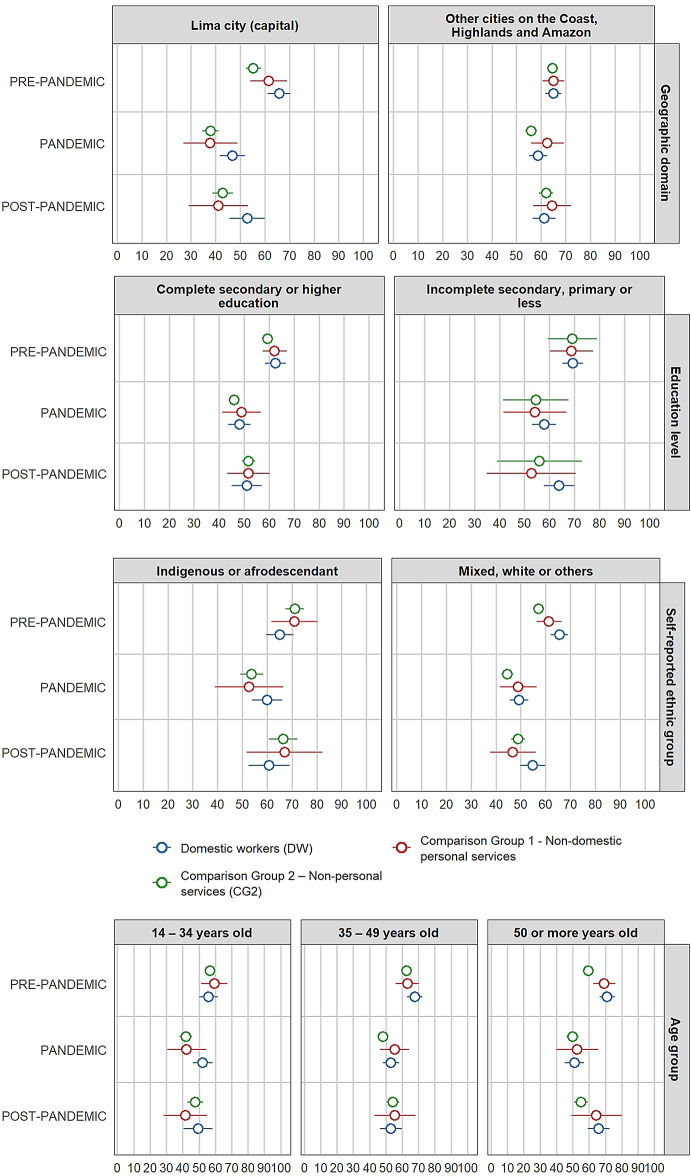

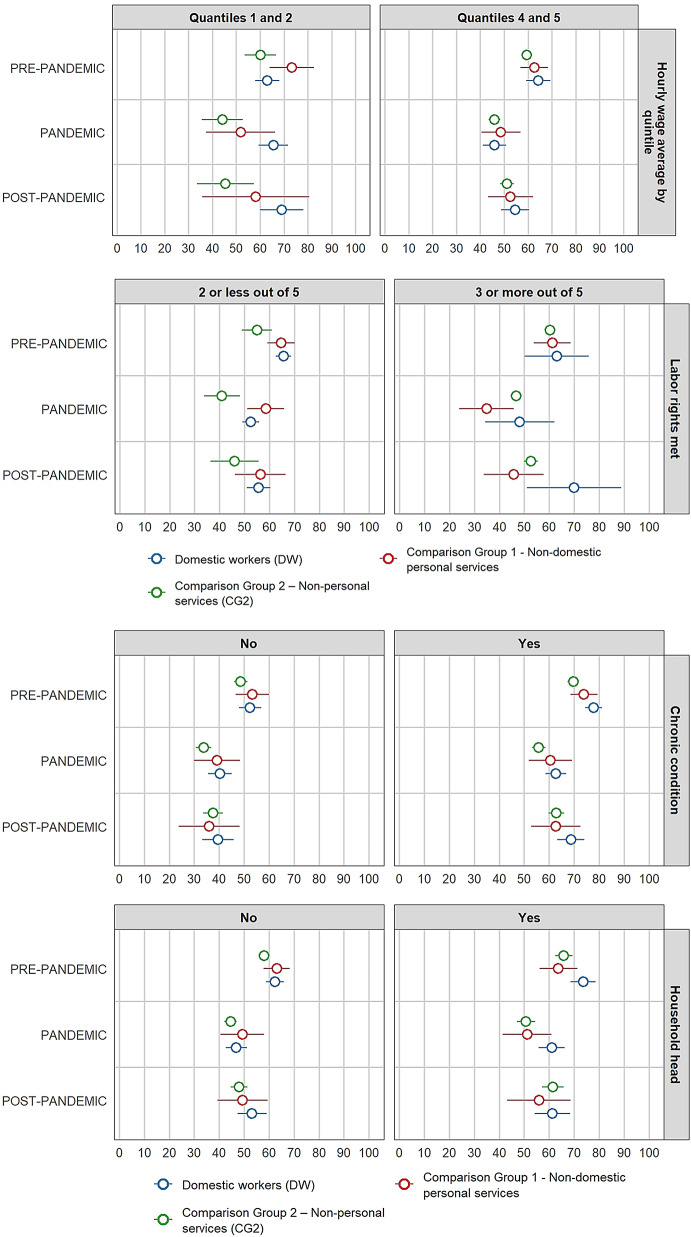
Stratified differences in proportions by individual characteristics on illness symptoms between female paid domestic workers and comparison groups per period *(in percentages)*

Figure 2 shows illustrates the stratified proportion differences by individual characteristics on illness symptoms between domestic workers and comparison groups across each period. According to the results, the gap in illness symptoms between domestic workers and comparison group 1 is statistically equal to zero for all 51 possible combinations of periods and categories of individual characteristics.

At the baseline (pre-pandemic), mean differences between domestic workers and comparison group 2, stratified by geographic domain, self-identified ethnic group, age group, access to labour rights, and chronic condition, were statistically significant (95% CI). The gaps indicate a higher proportion of females with illness symptoms among domestic workers compared with comparison group 2. They are statistically significant for those living in Lima (10.5 pp.), females self-identifying as mixed or white (8.5 pp.), females aged 50 years or more (11.7 pp.), those with access to maximum two (out of five) labour rights (10.7 pp.), and those reporting a chronic condition (8.1 pp.).

During the pandemic period, differences in proportions between domestic workers and comparison group 2, stratified by geographic domain, age group, number of labour rights met, and chronic condition, remain statistically significant (95% CI) as in the pre-pandemic period. Compared with pre-pandemic levels, the gap widened during the pandemic among domestic workers living outside Lima, those with lower education, and those self-identifying as Indigenous or Afro-descendant. However, these differences were not statistically significant. In contrast, the gaps between domestic workers and comparison group 2 are smaller than in the pre-pandemic period for those living in Lima (8.9 pp.) and for those reporting a chronic condition (6.8 pp.). Among domestic workers and females from comparison group 2 aged 50 years and above, the gap decreases and is no longer statistically significant, whereas the gap for those aged 14–34 years increases compared with the pre-pandemic period and becomes statistically significant during the pandemic (10.4 pp.). Conversely, the illness symptoms gap for those with fewer labour rights increases (11.5 pp.). Additionally, the difference between both groups among those females who are heads of household becomes statistically significant (10.4 pp. at 95% CI).

In the post-pandemic period, the differences in proportions between domestic workers and comparison group 2, stratified by self-identified ethnic group and hourly wage quintile, are statistically significant (95% CI). Among females self-identifying as mixed or white, the gap in illness symptoms is 5.8 pp. For the first time, the stratified difference by wage quintile (first and second quintiles) becomes statistically significant, representing one of the largest gaps in the study (23.6 pp.).

Supplemental File 5 contains the differences in proportions on illness symptoms between domestic workers and comparison groups per period, including Wald test results. Supplemental File 6 includes the stratified differences by individual characteristics per period.

### Healthcare seeking behaviour

**Fig. 3:**
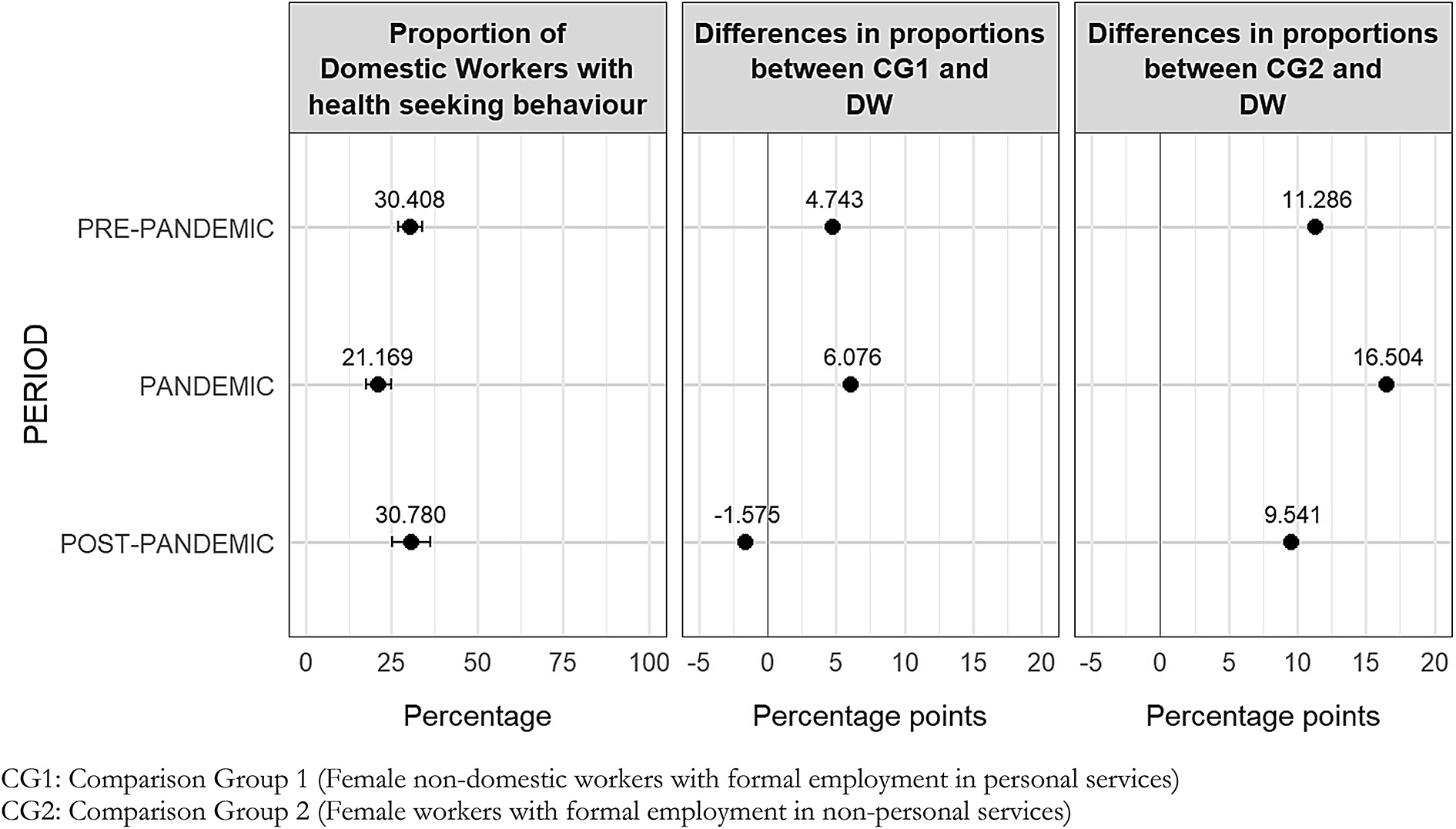
Differences on healthcare-seeking behaviour between female paid domestic workers and comparison groups by period *(proportions presented on percentage scale)*

Figure 3 presents the differences in healthcare-seeking behaviour between domestic workers and comparison groups before, during, and after the COVID-19 pandemic. In the pre-pandemic period, 30.4% of domestic workers sought healthcare when experiencing illness symptoms [95% CI: 26.8–34.0%], a proportion that is statistically similar to that of comparison group 1 [95% CI: 29.3–41.0%] but lower than that of comparison group 2 [95% CI: 39.6–43.8%]. The gap between domestic workers and comparison group 2 is 11.3% points, which is statistically significant [95% CI].

During the pandemic period, the healthcare seeking behaviour outcome among domestic workers was 21.2% [95% CI: 17.6% − 24.8%], and no statistical difference was found when comparing with the comparison group 1 [95% CI: 20.1% − 34.4%]. The proportion of females of comparison group 2 who sought healthcare attention [95% CI: 34.9% − 40.5%] was significantly higher than that of domestic workers, and the gap (16.5 pp.) increased compared to pre-pandemic.

In the post-pandemic period, the proportion of females with healthcare-seeking behaviour returned to levels very close to those observed in pre-pandemic for all groups. Among domestic workers, 30.8% [95% CI: 25.3–36.3%] sought healthcare, a proportion statistically similar to that of comparison group 1 [95% CI: 20.2–38.2%] but still significantly lower than comparison group 2 [95% CI: 37.0–43.6%]. The gap between domestic workers and comparison group 2 is 9.5% points, smaller than in previous periods.

**Fig. 4:**
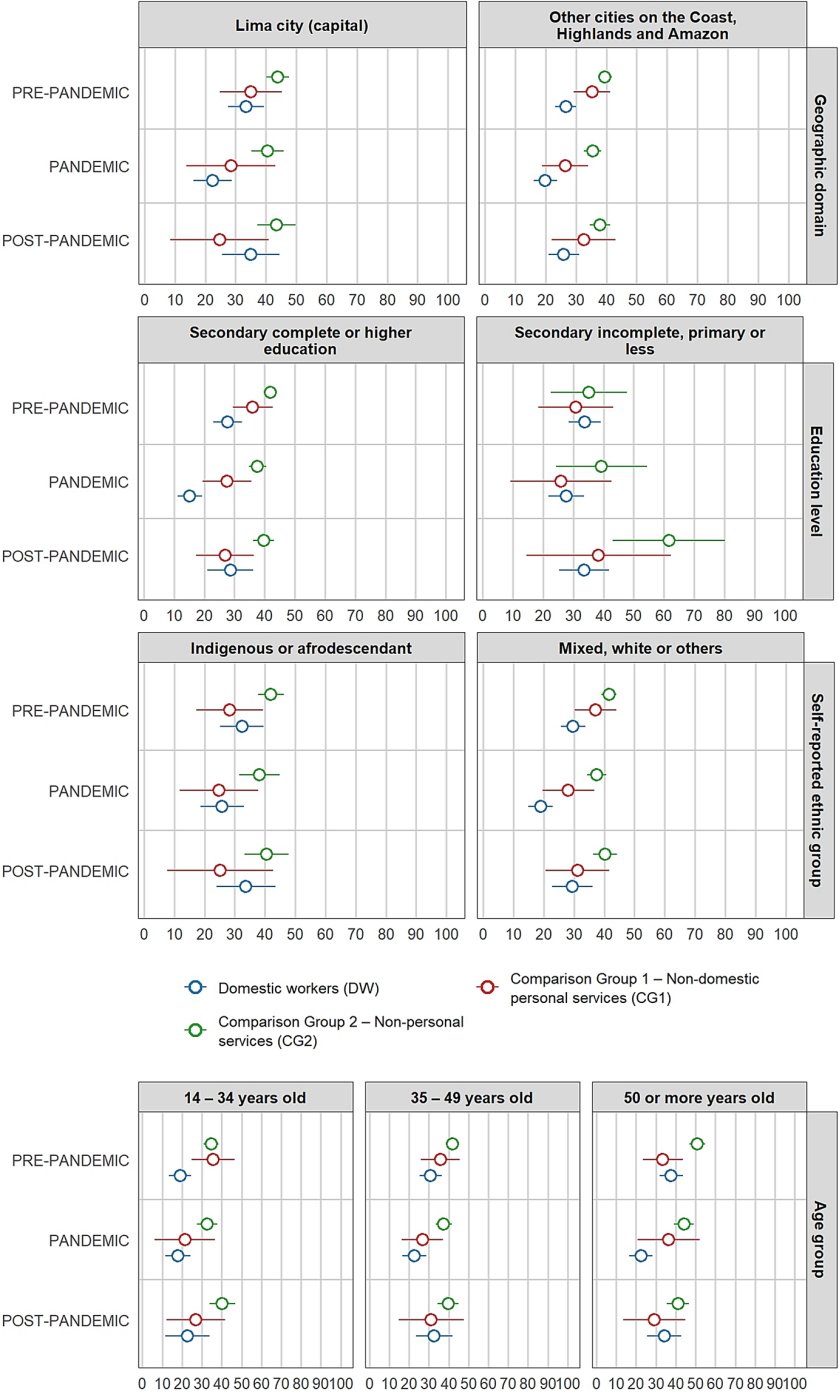

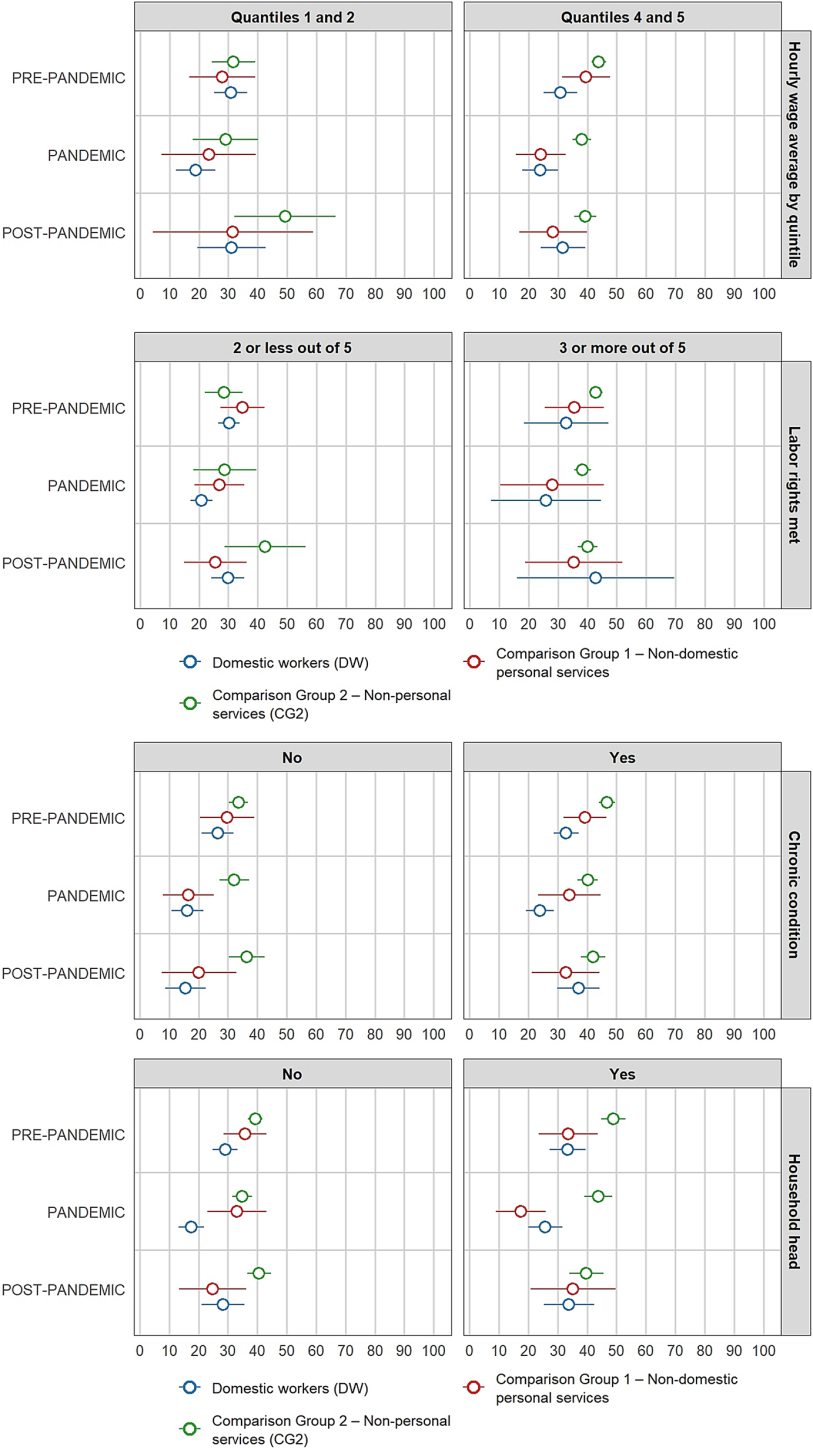
Stratified differences in proportions by individual characteristics in healthcare-seeking behaviour between female paid domestic workers and comparison groups per period *(in percentages)*

Figure 4 presents stratified differences in healthcare-seeking behaviour between domestic workers and the comparison groups across periods. The gap between domestic workers and comparison group 1 is statistically equal to zero for 48 of the 51 differences calculated across all possible paired combinations, periods, and categories of individual characteristics. The only statistically significant gaps are those by age group in the pre-pandemic period, and by education level and head-of-household status during the pandemic period. However, these differences are no longer statistically significant in the subsequent periods.

At the baseline (pre-pandemic), differences in healthcare-seeking behaviour between domestic workers and comparison group 2, stratified by geographic domain (Lima: − 10.4 pp.; other cities: − 12.9 pp.) and education level (completed secondary or higher education: − 14.2 pp.), are statistically significant (95% CI). Similar significant gaps are found when stratified by self-identified ethnic group (mixed or white: − 11.9 pp.), age group (14–34 years: − 15.6 pp.; 35–49 years: − 10.9 pp.; 50 + years: − 13.0 pp.), hourly wage quintiles (fourth and fifth quintiles: − 13.1 pp.), chronic condition (–13.9 pp.), and head-of-household status (–15.5 pp.). Overall, in the pre-pandemic period, 11 out of 17 stratified differences show a statistically significant lower prevalence of healthcare-seeking behaviour among domestic workers compared with comparison group 2.

During the pandemic, differences in proportions between domestic workers and comparison group 2, stratified by geographic domain (Lima: − 18.0 pp.; other cities: − 15.6 pp.) and education level (completed secondary or higher education: − 22.4 pp.), remain statistically significant (95% CI) and are larger (in absolute terms) than in the pre-pandemic period. Similar significant gaps are observed when stratified by self-identified ethnic group (mixed or white: − 18.5 pp.), age group (35–49 years: − 14.8 pp.; 50 + years: − 21.6 pp.), hourly wage quintiles (fourth and fifth quintiles: − 14.2 pp.), chronic condition (–16.2 pp.), and head-of-household status (–18.1 pp.). Ten of the seventeen stratified mean differences that are statistically significant between domestic workers and comparison group 2 are wider than in the pre-pandemic period. Additionally, the gaps between both groups increased during the pandemic for those domestic workers and females from comparison group 2 self-identifying as Indigenous, those with more limited access to labour rights, and those with the lowest hourly wages, although these differences are not statistically significant.

In the post-pandemic period, the differences between domestic workers and comparison group 2, stratified by geographic domain (other cities: − 11.9 pp.) and education level (completed secondary or higher education: − 11.0 pp.), remain statistically significant (95% CI) but are smaller (in absolute terms) than in pre-pandemic and pandemic periods. However, stratified differences by education level (incomplete secondary or less: − 28.1 pp.) and age group (14–34 years: − 17.5 pp.) are also statistically significant and larger than in previous periods.

Supplemental File 5 shows the differences in proportions on healthcare seeking behaviour between domestic workers and comparison groups per period, and corresponding Wald test results. Supplementary File 7 includes the stratified differences by individual characteristics per period.

## Discussion

Literature on the effect of the pandemic on health outcomes among domestic workers in Peru remains scarce. The few quantitative studies available are based on small-scale surveys conducted during the first two years of the pandemic.This study focused on analysing the disparities’ changes on i) illness symptoms and ii) healthcare seeking behaviour between female domestic workers and females with formal employment within the service sector before, during and after the COVID-19 pandemic. 

 Our analysis indicates that female paid domestic workers consistently reported a higher proportion of illness symptoms compared with females with in formal non-domestic personal service roles (comparison group 1) and those in formal non-personal service employment (comparison group 2) across the pre-pandemic, pandemic and post-pandemic periods. Notably, only differences observed in relation to comparison group 2 were statistically significant as the 95% confidence intervals for these differences did not include zero across the periods, -5.7 percentage points (pp.) before the pandemic, -5.9 pp. during the pandemic and -4.6 pp. after the pandemic. 

Differences in the proportions of illness symptoms between domestic workers and formal workers in non-personal services changed according to individual characteristics across periods. The differences in illness symptoms between groups were statistically significant during the pandemic for younger women, those with more limited access to labour rights, and those who were household heads, reflecting greater disparities than pre-pandemic. Significant gaps were also observed before and during the pandemic for women living in Lima and for those with chronic conditions, but these differences narrowed during the pandemic compared to pre-pandemic levels. Although most stratified differences declined in the post-pandemic period, there were significant gaps between both groups during and after the pandemic among females with the lowest wages (first and second quantiles) compared to the pre-pandemic period.

Although the literature review did not identify research comparing health inequalities between domestic workers and formal workers, the elevated incidence of illness symptoms among domestic workers aligns with findings from priors studies examining the impact of the COVID-19 pandemic impacts on this group. Recent research of 356 domestic workers in Bulgaria, India, Mexico, Peru and Thailand found that health and safety risks increased between 2020 and 2021 due to the informal nature of their work. In fact, 50% of respondents reported increased exposure to COVID-19 in their workplaces [22]. A study conducted in Hong Kong found that domestic workers were between 1.81 and 4.56 times more likely to be exposed to SARS-CoV-2 than other vulnerable populations because they were forced by the government to live with their employers [23]. Another study, based on telephone interviews with 2,650 domestic workers in 14 Latin American countries in 2020, found that 56% did not receive personal protective equipment at their workplace [5]. The nature of domestic work placed these women at high risk of exposure, leaving them dependent on their employers’ willingness to enforce health and safety measures during the pandemic.

Recent reports in the grey literature examining the impact of the COVID-19 pandemic on domestic workers in Peru indicate that a significant number of domestic workers consented to employer requests to reside at their workplaces, resulting in extended working hours and hightened vulnerability to exploitative conditions [14]. The pandemic also disrupted domestic workers’ transportation and mobility patterns, negatively affecting their family responsibilities, household budgets, and work schedules [15],which could affect their mental well-being. Furthermore, a report about the psychosocial risk to 100 domestic workers in Lima during the pandemic revealed that 43% and 37% of those between 31 and 45 years old had high exposure to burnout and stress, respectively [16].

For the second outcome, healthcare-seeking behaviour, domestic workers showed a lower proportion than formal workers in non-domestic personal services (comparison group 1) before and during the pandemic, although differences were not statistically significant. However, gaps with formal workers in non-personal services (comparison group 2) were statistically significant in all three periods, with the most considerable differences observed during the pandemic (16.5 pp.).

As with the first outcome, gaps in healthcare-seeking behaviour between domestic workers and comparison group 2 varied by individual characteristics across periods. In the pre-pandemic period, significant differences were observed across geographic domain, education level (complete secondary or higher), ethnic self-identification (mixed or white), hourly wage quintile (upper quintiles), all age groups, chronic conditions, and household head status. Compared with pre-pandemic levels, these stratified gaps widened during the pandemic. In the post-pandemic period, overall differences in healthcare seeking between domestic workers and comparison group 2 returned to pre-pandemic levels. Notably, gaps for women with lower education and for younger women were significantly larger in the post-pandemic period compared with pre-pandemic levels.

These findings align with previous studies about the pandemic's effects on domestic workers. For instance, research conducted in the United Kingdom found that one of the most significant effects of the pandemic was disruption to health care, particularly among women and low-skilled, low-income groups such as domestic workers [24]. A longitudinal study in an Indian city that followed a sample of 292 domestic workers during the first six months of the pandemic found that the proportion of women experiencing symptoms remained stable over time, but the proportion seeking medical care decreased significantly [25]. Similarly, telephone interviews with 40 domestic workers in three Indian cities found that many women discontinued treatment for reproductive health issues and chronic diseases during the pandemic due to lack of financial support and resources [26].

Evidence from high-income countries shows that older individuals, non-white ethnic minorities, and those from lower social classes are more likely to discontinue medical treatments and stop seeking healthcare [24]. In Peru, a study using administrative data on healthcare visits documented a reduction in service provision at the onset of the pandemic, due to the country’s fragmented health system and the shortage of specialised health personnel [27]. The same study cited two literature reviews covering data from over 40 countries, both of which reported significant reductions in healthcare use during the first year of the pandemic—findings that are consistent with our results. Similarly, using data from ENAHO, a research found that living in urban areas of the Amazon increased the likelihood of not using formal healthcare services, while individuals over 50 years old and those with higher education were more likely to seek care, which aligns with our findings [28].

Previous studies indicate that the pandemic deepened pre-existing economic and social inequalities in Peru, disproportionately affecting vulnerable populations—particularly informal, low-skilled workers with limited access to social protection, including domestic workers [29]. For example, a study on the persistent effects of COVID-19 on labour outcomes found that average labour income fell by 76% for informal workers without secondary education, compared with a 40.8% reduction for formal, more educated workers [11]. This finding is consistent with evidence from Ethiopia, where pre-pandemic data indicated that households headed by domestic workers had the highest probability of persistent poverty compared with households headed by more educated individuals in non-domestic occupations [30].

These patterns highlight the structural nature of the observed inequalities. In Peru, persistently high levels of labour informality are interlinked with ethnic and class-based discrimination and the fragmentation of social protection systems. Together, these factors restrict domestic workers’ ability to exercise fundamental rights, including access to healthcare and social security, with direct implications for their health outcomes. Addressing these inequalities requires integrated policies that go beyond improving service availability to tackle the root causes of exclusion, promote labour market formalisation, and guarantee universal and portable social security coverage.

### Strengths, limitations and future research

This study has several strengths. First, it draws on six years of national data from Peru—the country most severely affected by the pandemic worldwide—allowing for assessing patterns on health disparities before, during, and after the COVID-19 pandemic. Second, it focuses on female paid domestic workers, an occupational group that has been historically underrepresented in health inequality research and disproportionately affected by COVID-19 globally, and compares them with two well-defined reference groups, thereby strengthening the interpretability of the observed differences. Third, the analysis stratifies results by key sociodemographic and labour characteristics (e.g., geographic domain, education, ethnicity, age, income, chronic condition, household head status), enabling the identification of subgroups in which disparities are larger or were amplified during the pandemic. Fourth, the findings contribute to the ongoing discussion about the benefits and strategies of increasing formal employment among domestic workers, particularly in light of the 2020 legislation granting them labour rights equivalent to workers with formal employment.

The study also has limitations. First, as it relies on cross-sectional survey data aggregated by period, the study cannot establish causal relationships between occupational group and health outcomes. Second, temporal differences should not be interpreted as trends, as the results were not adjusted for broader macroeconomic or policy changes. Third, the findings cannot be generalized to all racialized or geographically marginalized groups, as these variables were examined only within the domestic worker population. Fourth, the study assessed only two health outcomes. Further research on domestic workers’ health could benefit from ad hoc surveys specifically designed for this population, considering the particular nature of their work. Such surveys should include a broader range of questions on their health status, working conditions—such as workload, job stability, access to social protection, exposure to occupational hazards, and employer–employee relationships—and allow for better measurement of intermediate social determinants of health, including housing quality, food security, access to healthcare, and social support networks.

### Policy implications

Disparities in health outcomes between domestic workers and women with formal employment in non-personal services underscore the importance of addressing systemic labour informality and developing effective, inclusive social protection systems to achieve health-related Sustainable Development Goals (SDGs).

The COVID-19 pandemic exposed deep-seated structural weaknesses in Peru’s health system, including fragmentation, chronic underinvestment, and limited government coordination, all of which undermined the national response [31]. The lack of resilient healthcare infrastructure contributed to the adoption of strict lockdown measures; however, cities with weaker systems collapsed under pressure. Ineffective management and poor integration between public and private healthcare providers further deepened the crisis. These systemic challenges are compounded by long-standing inequities linked to ethnic and class discrimination, which disproportionately affect workers in informal and precarious jobs—particularly women in domestic service. Addressing these intertwined issues requires rethinking development models and strengthening global health reporting mechanisms to capture and address the structural determinants of inequality.

Differences in health-seeking behaviour are not merely the result of individual choice or biological predisposition but are shaped by intersecting social, economic, and cultural determinants. While genetic and environmental factors may largely influence illness symptoms may, public policy can and should address the institutional barriers that limit healthcare access for domestic workers. These include not only expanding social security coverage but also ensuring equitable distribution of resources, culturally appropriate health services, and legal protections that dismantle discriminatory practices in the labour market.

Therefore, policy efforts should extend beyond isolated interventions and become form part of a broader strategy to address systemic informality, reduce ethnic and class-based discrimination, and integrate fragmented social protection systems. This could involve formalisation incentives, legal reforms to guarantee universal portability of health coverage across public providers, and targeted outreach programmes for groups facing multiple vulnerabilities, such as ethnic minorities, those with lower salaries, and those with chronic conditions.

## Conclusion

This study shows that female paid domestic workers consistently experience a higher prevalence of illness symptoms and lower healthcare-seeking behaviour than women in formal non-personal service jobs, with disparities peaking during the pandemic and narrowing thereafter. The persistence of these gaps—particularly among women with the lowest salaries, lower education, and younger age—underscores the role of systemic factors that extend beyond the health sector.

These findings point to the enduring influence of structural determinants of health inequalities in Peru, including systemic labour informality, ethnic and class-based discrimination, and the fragmentation of social protection systems. Addressing these deep-rooted issues requires more than isolated measures: it demands comprehensive strategies that promote formalisation, guarantee universal and portable social security coverage, and ensure equitable access to culturally appropriate healthcare. Sustained public efforts in these areas are crucial to reducing health disparities, safeguarding the health and well-being of domestic workers, and make meaningful progress towards Sustainable Development Goal 3 (SDG3).

## Supplementary Information

Below is the link to the electronic supplementary material.

Supplementary Material 1

## Data Availability

All original datasets are available from the website of the National Institute of Statistics and Informatics of Peru: [https://proyectos.inei.gob.pe/microdatos/](https:/proyectos.inei.gob.pe/microdatos) .
