# Supplementary material for: Assessing health disparities faced by female paid domestic workers in Peru before, during, and after the COVID-19 pandemic
Source: Int J Equity Health. 2025 Nov 26;24:335. doi: 10.1186/s12939-025-02617-w (PMC12661671; doi:10.1186/s12939-025-02617-w)
Supplement: Supplementary file 1 — Supplementary Material 1 [file 12939_2025_2617_MOESM1_ESM.docx]

Assessing Health Disparities Faced by Female Paid Domestic Workers in Peru before, during, and after the COVID-19 Pandemic

(Supplementary materials)

**Authors:**

Vera-Tudela David^1^, Cárdenas Maria Kathia^2^, Díaz Ramón^1^, Meaney Christopher^3^, Lazo-Porras María^2^, Cruzado Viviana^4^, Gupta Archna^3,5^, Tenorio-Mucha Janeth^2^

**Affiliations:**

1. Universidad Peruana Cayetano Heredia (Lima, Peru)
2. CRONICAS Center of Excellence in Chronic Diseases, Universidad Peruana Cayetano Heredia (Lima, Peru)
3. Department of Family and Community Medicine, University of Toronto (Toronto, Canada)
4. Pontificia Universidad Católica del Perú (Lima, Peru)
5. Upstream Lab, MAP Centre of Urban Health Solutions, Li Ka Shing Knowledge Institute, St. Michael’s Hospital (Toronto, Canada)

**Corresponding author:**

David Vera-Tudela

david.vera.tudela@upch.pe

**SUPPLEMENTARY FILE 1**

**Definition and composition of Comparison Groups**

Both comparison groups only include females with formal employment in any economic activity within the service sector (excluding domestic work) and are either: i) employees with social security paid by their employers, or ii) independent workers registered with the National Tax Authority.

According to the International Standard Industrial Classification of All Economic Activities (ISIC), Revision 4, domestic work is included in the division 97 “Activities of households as employers of domestic personnel” of section “T - Activities of households as employers; undifferentiated goods- and services-producing activities of households for own use”.

As domestic work is considered within personal services activities, the Comparison Group 1 (CG1) are female formal workers from two ISIC’s sections (I and S), which represent personal service activities, those focused on individuals and their specific needs, excluding domestic work. Personal services activities include the following:

1. **“Accommodations and food services”** (section I)
2. **“Washing and (dry-)cleaning of textile and fur products”** (section S: class 9601)
   include activities such as laundry services for garments, linens, and carpets.
3. **“Hairdressing and other beauty treatment”** (section S: class 9602,)
   Includes haircuts, hairstyling, manicures, pedicures, and facial treatments.
4. **“Other personal service activities (OPS)”**, including “Funeral and related activities” (section S: class 9603) and “other personal service activities not classified” (section S: class 9604.

The second comparison group (CG2) are female formal workers engaged in non-personal service activities, activities focused on organisations or broader functional needs, often involving goods or systems rather than direct individual benefits. This group include 12 ISIC sections, which can be grouped into six categories based on their similarity and the distribution of female workers engaged in these activities:

1. **“Transportation, storage and communication”**, including “Transportation and storage” (section H) and “Information and communications” (section J).
2. **“Financial and insurance, real estate, professional, scientific and administrative support activities”.** It includes “Financial and insurance activities” (section K), “Real estate activities” (section L), “Professional, scientific and technical activities” (section M) and “Administrative and support service activities” (section N).
3. **“Public administration”.** “Public administration and defence” (section O) and “Activities of extraterritorial organizations and bodies” (section U).
4. **“Education”** (section P)
5. **“Health and social work”** (section Q)
6. **“Other non-personal service activities (NPS)”**. These activities evolve “Arts, sports and recreation” (section R), “Electricity, gas, steam and air conditioning supply” (section D), “Water supply; sewerage, waste management and remediation activities (section E), and the group of “Association activities” (from section S).

**SUPPLEMENTARY FILE 2**

**Variables definitions**

| **Outcomes** | **ENAHO’s variable** | **ENAHO’s question** | **Categories from the ENAHO's question** | **Definition of transformed variable** |
| --- | --- | --- | --- | --- |
| **Illness Symptoms** | p402 | *In the last 4 weeks, from … to … did you experience any of the following conditions?:* | 1= Symptom or discomfort (e.g., cough, headache, fever, nausea)  2=Illness (e.g., flu, colitis, etc.)  3=Relapse of a chronic illness  4=Accident  5=Did not experience any illness, symptom, relapse, or accident  6=COVID-19 symptom (e.g., fever, dry cough, shortness of breath) | The variable has been transformed and renamed *enfermedad4ultgsem* based on responses to question p402. The transformed outcome is coded as 1 if p402 takes any value from options 1, 2, 3, 4, or 6, and as 0 if p402 takes the value 5. Observations with missing values were excluded from the construction of this variable. |
| **Healthcare seeking behaviour** | p403 | [Only for those who responded options 1, 2, 3, 4, 6 in p402]  *Where did you go to seek care for this illness, symptom, discomfort, and/or accident?*  *(Multiple responses allowed)* | 1=Public Primary healthcare post (MINSA)  2=Public Primary healthcare center (MINSA)  3=Community primary healthcare facility (CLAS)  4=Social security primary healthcare facility (ESSALUD)  5=Public Hospital (MINSA)  6=Social Security hospital (ESSALUD)  7=Armed Forces and/or National Police hospital  8=Private medical practice  9=Private clinic  10=Pharmacy or drugstore  11=At the patient’s home  13=Other ________  (Specify)  14=Did not seek care | The variable has been transformed and renamed *buscoatencion_enf4ultsem* based on responses to question p403. This new variable was created to identify whether individuals with illness symptoms in the past four weeks sought medical care.  The variable was coded as 1 if the respondent reported options 1 to 9 from p403. It was coded as 0 if no care was sought (options from 10 to 14).  Missing or invalid responses were excluded.  Value labels were assigned as follows:  1 = Sought medical attention  0 = Did not seek medical attention |

| **Individual characteristics** | **ENAHO’s variable name** | **ENAHO’s**  **question** | **Categories of ENAHO's question** | **Definition of transformed variable** |
| --- | --- | --- | --- | --- |
| **Age group** | p208a | *How old are you?* | Open question  (age in years) | The age group variable has four categories which are based on the aggregation of the original categories from question p208a:  Age Group 1 (14 to 19): if p208a >= 14 and <= 19  Age Group 2 (20 to 34): if p208a >= 20 and <= 34  Age Group 3 (35 to 49): if p208a >= 35 and <= 49  Age group 4 (50 and more): if p208a >= 50 |
| **Geographic domain** | estrato | Population size of the Primary Sample Unit (settlement) where the interviewed household belongs acccording to the ENAHO's sample design | 1. 500 000 or more inhabitants  2. From 100 000 to 499 999 inhabitants  3. From 50 000 to 99 999 inhabitants  4. From 20 000 to 49 999 inhabitants  5. From 2 000 to 19 999 inhabitants  6. From 500 to 1 999 inhabitants  7. Complex rural settlements  8. Simple rural settlements | According with Peruvian's Statistics Office, Urban areas include settlements with 2000 inhabitants or more (estrato >= 1 and estrato <=5), and Rural areas include settlements with less than 2000 inhabitants (estrato>=6 and estrato<=8)  Geographic domain 1 (urban coast) if estrato=urban area and domain>=1 and domain <=3  Geographic domain 2 (urban highlands) if estrato=urban area and domain>=4 and domain <=6  Geographic domain 3 (urban amazon) if estrato=urban area and domain=7  Geographic domain 4 (Lima city) if domain =8 |
|  | dominio | Geographic area where the interviewed household is located | 1.North coast  2.Central coast  3.South coast  4.North Highlands  5.Central Highlands  6.South Highlands  7.Amazon  8.Lima City |  |
| **Education level** | p301a | *Which is your highest educational level attaintment?* | 1. None  2. Pre-school  3. Incomplete primary  4. Complete primary  5. Incomplete secondary  6. Complete secondary  7. Incomplete higher education (non-university institution)  8. Higher education degree (non-university institution)  9. Incomplete higher education (university)  10. Higher education degree (university)  11. Post graduate (master/PhD)  12. Special education needs education | The education level variable has four categories which are based on the aggregation of the original categories from question p301a:  1-4, 12: Complete primary education or less  5: Incomplete secondary education  7-11: Any higher level education |
| **Self-reported ethnic group** | p558c | *According to your ancestors and costumes, which ethnic group do you belong?* | 1. Quechua  2. Aymara  3. Amazon tribe  4. Afrodescendant  5. White  6. Mixed ("Mestizo")  7. Other  8. No answer  9. Another indigenous group | The variable was transformed based on responses to question p558c as follows:  Ethnic group 1 (indigenous): p558c>=1 and p558c<=3 or p558c=9  Ethnic group 2 (Afrodescendants): p558c=4  Ethnic group 3 (White/others): p558c=5 or p558c=7 or p558c=8  Ethnic group 4 (Mixed): p558c=6 |
| **Hourly wage average by quintile** | inglab1 | *How much did you earn in your main job over the last 12 months?* | Open numeric response | inglab1 was generated as the annual income from the main job for employed individuals. It was divided by 12 to obtain a monthly wage and then by 4 to calculate the weekly wage.  Hourly wage (ing1hora) was calculated by dividing the weekly wage by the number of hours worked in the last week . The variable was adjusted by applying the currency exchange rate to convert values from soles to US dollars. Subsequently, the sample weights were distributed into quintiles, and the hourly wage average was calculated for each quintile. |
|  |  |  |  |  |
|  |  |  |  |  |
|  |  |  |  |  |
|  |  |  |  |  |
|  |  |  |  |  |
|  |  |  |  |  |
|  |  |  |  |  |
|  |  |  |  |  |
| **Access to labor rights** | p511a | *What type of contract do you have?* | 1: Permanent contract  2: Fixed-term contract  3: Trial period  4: Internship or youth employment program  5: Independent contractor  6: Special Administrative Contracting Regime  7: No contract  8: Other | The variable was transformed based on responses to question p511a as follows considering:  0 (without labor contract) if p511a = 7 (no contract)  1 (with labor contract) if p511a adopts any other value |
|  | p558a | *What pension system are you affiliated with?* | 1: Private pension system (AFP)  2: National Pension System (Law 19990)  3: National Pension System (Law 20530)  4: Other  5: Not affiliated | The second condition (afil_SP) for labor formality is affiliation to the pension system:  1(is affiliated) if p558a = 1, 2, 3, or 4  0 (if not) if p558a = 5 .  This classification is based on reported affiliation to either public, semi-public, special, or private pension systems. |
|  | p419a1 | *Are you covered by the social security (Essalud) paid by your employer?* | 1: Yes  2: No | The third condition for labor formality is ss_emp_essalud, this variable was generated to identify affiliation to social security (EsSalud) through employer contribution.  1 (affiliated to the social security paid by the employer) if p419a1= 1  0 (otherwise) if p419a1 = 2 |
|  | p5441a | *Did you receive the first anually allowance? (July)* | 1: Yes  2: No | The fourth and fifth conditions for labor formality are based on the receipt of legally mandated annual allowances. According to Peruvian labor regulations, every dependent worker must receive one allowance in July (National Holidays) and another in December (Christmas), each equivalent to one month’s salary.  1 (received the July allowance) if p5441a = 1  0 (if not) if p5541a = 2  1 (received the December allowance) if p5442a = 1  0 (if not) if p5542a = 2 |
|  | p5442a | *Did you receive the second anually allowance? (december)* | 1: Yes  2: No |  |
|  | p511a  p558a  p419a1  p5441a  p5442a | *Number of labor rights* | 5 = Access to five labor rights  4 = Access to four labor rights  3 = Access to three labor rights  2 = Access to two labor rights  1 = Access to one labor rights  0 = Access to none labor rights. | The variable *Ncrit_form5* was constructed as an index that counts how many of five key labor rights are granted to formal workers mention above.  It was generated using the rsum() function across the following binary variables:  - p511a: has a contract  - p558a: affiliated with a pension system  - p419a1: has EsSalud provided by the employer  - p5441a: received National Holidays allowance  - p5442a: received Christmas allowance  The resulting index ranges from 0 to 5 and reflects the number of entitled labor rights accessed by the worker |
| **Chronic disease Condition** | p401 | *¿Do you suffer from any chronic disease? Arthritis, hypertension, asthma, rheumatism, diabetes, tuberculosis, HIV,high cholesterol, etc.* | 1: Yes  2: No | The variable enfcronica was created to identify if the individual has a chronic disease. Its coded 1 if they have a chronic disease, and 0 otherwise. |
| **Household head status** | p203 | *What is your relationship to the head of household?* | Head of household: 1  Spouse/partner: 2  Son/daughter or stepson/stepdaughter: 3  Son/daughter-in-law: 4  Grandchild: 5  Parents/in-laws: 6  Sibling: 11  Other relatives: 7  Domestic worker: 8  Pensioner: 9  Other non-relatives: 10 | The binary variable jefe was created to identify the household head. It takes the value 1 when the reported relationship to the household is "head" (p203 == 1), and 0 in all other cases. |

**SUPPLEMENTARY FILE 3**

**3.1 Occupied female in services related activities per period**

(% occupied female in service sector – weighted sample)

|  | **Activities** | **Pre-pandemic** | **Pandemic** | **Post-pandemic** | **All periods** | **Category** |
| --- | --- | --- | --- | --- | --- | --- |
| **Service sector** | | **100.0** | **100.0** | **100.0** | **100.0** | **Service sector** |
| Personal services activities | Domestic work | 11.9 | 11.5 | 11.8 | 11.7 | Domestic work activities |
|  | Accommodation and food service activities | 29.2 | 27.6 | 29.1 | 28.7 | Non-domestic personal services |
|  | Washing and (dry-)cleaning of textile and fur products | 2.2 | 2.6 | 2.2 | 2.3 |  |
|  | Hairdressing and other beauty treatment | 2.6 | 2.5 | 2.6 | 2.6 |  |
|  | Other personal services | 2.4 | 2.1 | 2.2 | 2.2 |  |
| Non-personal services activities | Transportation, storage and communications | 4.4 | 4.3 | 4.2 | 4.3 | Non-personal services activities |
|  | Financial and insurance, real estate, professional, scientific, and administrative support activities | 14.0 | 14.2 | 14.3 | 14.1 |  |
|  | Education | 13.8 | 13.0 | 12.7 | 13.2 |  |
|  | Human health and social work activities | 8.7 | 10.0 | 9.1 | 9.2 |  |
|  | Public administration and defence | 7.2 | 8.9 | 7.5 | 7.8 |  |
|  | Other non-personal services | 3.8 | 3.4 | 4.2 | 3.8 |  |

Economic activities grouped according to the Standard Industrial Classification of All Economic Activities (ISIC) v.4.0. Data source: ENAHO 2018 – 2023.

**3.2 Occupied female with formal employment in services related activities per period**

(% occupied female – weighted sample)

| **Category** | **Pre-pandemic** | **Pandemic** | **Post-pandemic** | **All periods** |
| --- | --- | --- | --- | --- |
| **Service sector** | 35.12 | 37.42 | 34.62 | 35.92 |
| **Domestic work** | 10.21 | 18.90 | 9.02 | 13.39 |
| **Non-domestic personal services activities** | 11.46 | 11.40 | 9.69 | 11.06 |
| **Non-personal services activities** | 59.75 | 62.95 | 59.07 | 60.87 |

Economic activities grouped according to the Standard Industrial Classification of All Economic Activities (ISIC) v.4.0. Data source: ENAHO 2018 – 2023.

**SUPPLEMENTARY FILE 4**

**Characterization of study population by period**

(% weighted sample)

| **Pre- Pandemic** | | | |
| --- | --- | --- | --- |
| **Characteristic** | **Domestic workers** | **CG1 - personal services** | **CG2 - non-personal services** |
|  |  |  |  |
| **Sample observations (weighted)** | 764 650 | 276 515 | 2 037 464 |
| **Sample observations (non weighted)** | 2,004 | 921 | 6,768 |
|  |  |  |  |
| **Age group** | | | |
| 14-19 | 6.07 | 1.77 | 0.68 |
| 20-34 | 21.81 | 35.58 | 35.39 |
| 35-49 | 40.78 | 38.01 | 38.33 |
| 50+ | 3134 | 24.83 | 25.59 |
| **Geographic domain** | | | |
| Coast | 24.44 | 20.39 | 19.5 |
| Highlands | 13.16 | 20.29 | 20.77 |
| Amazon | 7.34 | 8.66 | 7.11 |
| Lima city (capital) | 55.06 | 50.66 | 52.62 |
| **Education level** | | | |
| Complete primary or less | 26.07 | 8.55 | 1.25 |
| Incomplete secondary | 16.04 | 7.6 | 1.57 |
| Complete secondary | 38.32 | 35.62 | 11.06 |
| Higher education | 19.57 | 48.22 | 86.11 |
| **Self-reported ethnic group** | | | |
| Indigenous | 19.68 | 16.09 | 15.77 |
| Afrodescendant | 5.66 | 4.15 | 3.14 |
| White and others | 14.48 | 14.01 | 9.83 |
| Mixed background | 60.18 | 65.75 | 71.26 |
| **Hourly wage average by quintile (US$)** | | | |
| 1Q (lower income) | 0.26 | 0.22 | 0.22 |
| 2Q | 0.89 | 0.92 | 0.98 |
| 3Q | 1.48 | 1.49 | 1.48 |
| 4Q | 2.24 | 2.25 | 2.30 |
| 5Q (higher income) | 4.28 | 7.49 | 5.94 |
| **Number of labor rights met (out of 5)** | | | |
| 0 | 63.72 | 1.05 | 0.14 |
| 1 | 13.43 | 38.34 | 3.86 |
| 2 | 15.15 | 18.92 | 5.47 |
| 3 | 4.57 | 11.75 | 14.76 |
| 4 | 2.41 | 9.02 | 10.38 |
| 5 | 0.73 | 20.93 | 65.39 |
| **Chronic disease Condition** | | | |
| No | 48.65 | 51.62 | 47.18 |
| Yes | 51.35 | 48.38 | 52.82 |
| **Household head 1/** | | | |
| No | 70.58 | 72.29 | 77.44 |
| Yes | 29.42 | 27.71 | 22.56 |
|  | | | |

1/ For domestic workers: Live-out Domestic Workers only.

Data source: ENAHO 2018 – 2023.

| **Pandemic** | | | |
| --- | --- | --- | --- |
| **Characteristic** | **Domestic workers** | **CG1 - personal services** | **CG2 - non-personal services** |
|  |  |  |  |
| **Sample observations (weighted)** | 774 672 | 266 032 | 2 211 171 |
| **Sample observations**  **(non-weighted)** | 1,975 | 842 | 7,373 |
|  |  |  |  |
| **Age group** | | | |
| 14-19 | 7.29 | 5.58 | 0.41 |
| 20-34 | 23.9 | 38.9 | 37.54 |
| 35-49 | 38.28 | 36.22 | 37.31 |
| 50+ | 30.53 | 19.3 | 24.73 |
| **Geographic domain** | | | |
| Coast | 23.26 | 20.31 | 19.52 |
| Highlands | 12.8 | 22.75 | 20.53 |
| Amazon | 8.1 | 10.05 | 7.39 |
| Lima city (capital) | 55.84 | 46.9 | 52.56 |
| **Education level** | | | |
| Complete primary or less | 24.79 | 11.39 | 1.38 |
| Incomplete secondary | 17.12 | 10.16 | 1.41 |
| Complete secondary | 38.92 | 35.06 | 11.5 |
| Higher education | 19.16 | 43.39 | 85.7 |
| **Self-reported ethnic group** | | | |
| Indigenous | 20.59 | 21.34 | 16.25 |
| Afrodescendant | 6.35 | 4.01 | 3.47 |
| White and others | 16.19 | 12.38 | 9.44 |
| Mixed background | 56.87 | 62.27 | 70.84 |
| **Hourly wage average by quintile (US$)** | | | |
| 1Q (lower income) | 0.25 | 0.22 | 0.24 |
| 2Q | 0.80 | 0.81 | 0.87 |
| 3Q | 1.34 | 1.33 | 1.34 |
| 4Q | 1.97 | 2.02 | 2.04 |
| 5Q (higher income) | 4.06 | 6.17 | 5.36 |
| **Number of Labor rights met (out of 5)** | | | |
| 0 | 63.61 | 3.04 | 0.28 |
| 1 | 19.04 | 51.59 | 5.77 |
| 2 | 9.1 | 15.81 | 7.83 |
| 3 | 4.46 | 10.14 | 15.41 |
| 4 | 2.49 | 9.41 | 18.36 |
| 5 | 1.29 | 10.01 | 52.34 |
| **Chronic disease Condition** | | | |
| No | 46.86 | 49.47 | 43.2 |
| Yes | 53.14 | 50.53 | 56.8 |
| **Household head 1/** | | | |
| No | 60.11 | 69.29 | 72.38 |
| Yes | 39.89 | 30.71 | 27.62 |

1/ For domestic workers: Live-out Domestic Workers only.

Data source: ENAHO 2018 – 2023.

| **Post-Pandemic** | | | |
| --- | --- | --- | --- |
| **Characteristic** | **Domestic workers** | **CG1 - personal services** | **CG2 - non-personal services** |
|  |  |  |  |
| **Sample observations (weighted)** | 419 530 | 125 350 | 1 130 243 |
| **Sample observations (non weighted)** | 978 | 346 | 3,415 |
|  |  |  |  |
| **Age group** | | | |
| 14-19 | 5.06 | 2.37 | 0.44 |
| 20-34 | 24.98 | 40.04 | 39.02 |
| 35-49 | 34.99 | 34.74 | 35.66 |
| 50+ | 34.98 | 22.85 | 24.88 |
| **Geographic domain** | | | |
| Coast | 25.9 | 22.59 | 19.63 |
| Highlands | 9.6 | 15.72 | 19.95 |
| Amazon | 7.27 | 7.26 | 7 |
| Lima city (capital) | 57.23 | 54.43 | 53.42 |
| **Education level** | | | |
| Complete primary or less | 21.94 | 11.45 | 1.05 |
| Incomplete secondary | 18.92 | 8.01 | 1.77 |
| Complete secondary | 39.84 | 36.91 | 11.46 |
| Higher education | 19.3 | 43.62 | 85.71 |
| **Self-reported ethnic group** | | | |
| Indigenous | 21.56 | 17.33 | 14.42 |
| Afrodescendant | 6.39 | 7.72 | 2.6 |
| White and others | 18.36 | 11.37 | 10.91 |
| Mixed background | 53.7 | 63.58 | 72.06 |
| **Hourly wage average by quintile (US$)** | | | |
| 1Q (lower income) | 0.30 | 0.18 | 0.28 |
| 2Q | 0.79 | 0.68 | 0.85 |
| 3Q | 1.34 | 1.33 | 1.36 |
| 4Q | 2.06 | 2.00 | 2.07 |
| 5Q (higher income) | 4.01 | 5.64 | 5.48 |
| **Number of Labor rights met (out of 5)** | | | |
| 0 | 69 | 2.31 | 0.14 |
| 1 | 17.75 | 42.07 | 3.85 |
| 2 | 7.45 | 14.65 | 7.32 |
| 3 | 3.77 | 16.23 | 16.68 |
| 4 | 1.14 | 10.77 | 13.22 |
| 5 | 0.89 | 13.97 | 58.8 |
| **Chronic disease Condition** | | | |
| No | 41.85 | 40.29 | 43.11 |
| Yes | 58.15 | 59.71 | 56.89 |
| **Household head 1/** | | | |
| No | 57.41 | 61.45 | 70.94 |
| Yes | 42.59 | 38.55 | 29.06 |

1/ For domestic workers: Live-out Domestic Workers only.

Data source: ENAHO 2018 – 2023.

**SUPPLEMENTARY FILE 5**

**Outcomes values and Wald test results of differences between domestic workers and comparison groups by period**

**5.1 Illness symptoms - outcome values (percentage)**

| **Group** | **PRE-PANDEMIC** | | | | **PANDEMIC** | | | | **POST-PANDEMIC** | | | |
| --- | --- | --- | --- | --- | --- | --- | --- | --- | --- | --- | --- | --- |
|  | **Point estimate** | **Lower CI 95%** | **Upper CI 95%** | **OBS** | **Point estimate** | **Lower CI 95%** | **Upper CI 95%** | **OBS** | **Point estimate** | **Lower CI 95%** | **Upper CI 95%** | **OBS** |
| Domestic workers | 65.466 | 62.507 | 68.424 | 1,986 | 52.256 | 49.021 | 55.490 | 1,737 | 56.509 | 51.979 | 61.039 | 969 |
| CG1 - Non-domestic personal services | 63.284 | 58.936 | 67.632 | 910 | 49.970 | 43.446 | 56.494 | 600 | 51.950 | 44.373 | 59.526 | 343 |
| CG2 - Non-personal services | 59.771 | 57.916 | 61.626 | 6,731 | 46.343 | 44.238 | 48.447 | 5,800 | 51.948 | 49.317 | 54.580 | 3,383 |

Data source: ENAHO 2018 – 2023.

**5.2 Illness symptoms - Test results of differences in proportions (on percentage points scale)**

**(Comparison group - Domestic workers)**

| **Group** | **POINT ESTIMATE** | | | **F STATISTIC WALD TEST** | | | **PROB WALD TEST** | | |
| --- | --- | --- | --- | --- | --- | --- | --- | --- | --- |
|  | **P1** | **P2** | **P3** | **P1** | **P2** | **P3** | **P1** | **P2** | **P3** |
| CG1 - Non-domestic personal services | -2.181 | -2.286 | -4.559 | 0.660 | 0.370 | 0.960 | 0.417 | 0.543 | 0.327 |
| CG2 - Non-personal services | -5.695 | -5.913 | -4.560 | 9.890 | 9.350 | 3.080 | 0.002 | 0.002 | 0.080 |

P1: Pre-pandemic, P2: Pandemic, P3: Post-pandemic

Data source: ENAHO 2018 – 2023.

**5.3 Healthcare-seeking behaviour - outcome values (percentage)**

| **Group** | **PRE-PANDEMIC** | | | | **PANDEMIC** | | | | **POST-PANDEMIC** | | | |
| --- | --- | --- | --- | --- | --- | --- | --- | --- | --- | --- | --- | --- |
|  | **Point estimate** | **Lower CI 95%** | **Upper CI 95%** | **OBS** | **Point estimate** | **Lower CI 95%** | **Upper CI 95%** | **OBS** | **Point estimate** | **Lower CI 95%** | **Upper CI 95%** | **OBS** |
| Domestic workers | 30.408 | 26.787 | 34.030 | 1,296 | 21.169 | 17.576 | 24.762 | 981 | 30.780 | 25.256 | 36.304 | 563 |
| CG1 - Personal services | 35.151 | 29.289 | 41.014 | 561 | 27.245 | 20.051 | 34.440 | 326 | 29.205 | 20.223 | 38.187 | 200 |
| CG2 - Non-personal services | 41.694 | 39.583 | 43.806 | 4,141 | 37.673 | 34.853 | 40.492 | 2,933 | 40.321 | 36.994 | 43.648 | 1,911 |

Data source: ENAHO 2018 – 2023.

**5.4 Healthcare-seeking behaviour - Test results of differences in proportions (on percentage points scale)**

**(Comparison group - Domestic workers)**

| **Group** | **MEAN** | | | **F STATISTIC WALD TEST** | | | **PROB WALD TEST** | | |
| --- | --- | --- | --- | --- | --- | --- | --- | --- | --- |
|  | **P1** | **P2** | **P3** | **P1** | **P2** | **P3** | **P1** | **P2** | **P3** |
| CG1 - Personal services | 4.743 | 6.076 | -1.575 | 2.030 | 2.470 | 0.090 | 0.154 | 0.116 | 0.770 |
| CG2 - Non-personal services | 11.286 | 16.504 | 9.541 | 25.080 | 41.470 | 7.920 | 0.000 | 0.000 | 0.005 |

P1: Pre-pandemic, P2: Pandemic, P3: Post-pandemic

Data source: ENAHO 2018 – 2023.

**SUPPLEMENTARY MATERIAL 6**

**Illness Symptoms outcome values by individual characteristics of domestic workers and comparison groups by period (on percentage scale)**

**6.1 Period: Pre-pandemic (Illness symptoms)**

| **Variable** | **Categories** | **Group** | **Pre-pandemic** | | | |
| --- | --- | --- | --- | --- | --- | --- |
|  |  |  | **Point estimate** | **Lower CI 95%** | **Upper CI 95%** | **OBS** |
| **Geographic domain** | **Lima city (capital)** | Domestic workers | 65.847 | 61.177 | 70.516 | 554 |
|  |  | CG1 - Personal services | 61.575 | 54.084 | 69.066 | 191 |
|  |  | CG2 - Non-personal services | 55.389 | 52.367 | 58.412 | 1598 |
|  | **Other cities on the Coast, Highlands and Amazon** | Domestic workers | 65.003 | 61.704 | 68.302 | 1432 |
|  |  | CG1 - Personal services | 65.042 | 60.618 | 69.466 | 719 |
|  |  | CG2 - Non-personal services | 64.613 | 62.685 | 66.541 | 5133 |
| **Education level** | **Incomplete secondary, primary or less** | Domestic workers | 69.440 | 65.330 | 73.551 | 911 |
|  |  | CG1 - Personal services | 68.741 | 60.084 | 77.398 | 191 |
|  |  | CG2 - Non-personal services | 69.248 | 59.438 | 79.059 | 158 |
|  | **Complete secondary or higher education** | Domestic workers | 62.626 | 58.510 | 66.742 | 1075 |
|  |  | CG1 - Personal services | 62.235 | 57.387 | 67.084 | 719 |
|  |  | CG2 - Non-personal services | 59.494 | 57.616 | 61.371 | 6572 |
| **Self-reported ethnic group** | **Mixed, white or others** | Domestic workers | 65.593 | 62.180 | 69.007 | 1484 |
|  |  | CG1 - Personal services | 61.339 | 56.317 | 66.360 | 700 |
|  |  | CG2 - Non-personal services | 57.098 | 55.007 | 59.188 | 5362 |
|  | **Indigenous or afrodescendant** | Domestic workers | 65.091 | 59.530 | 70.651 | 502 |
|  |  | CG1 - Personal services | 71.064 | 61.771 | 80.357 | 210 |
|  |  | CG2 - Non-personal services | 71.148 | 67.475 | 74.820 | 1369 |
| **Hourly wage average by quintile** | **Quantiles 4**  **and 5** | Domestic workers | 64.401 | 59.370 | 69.432 | 647 |
|  |  | CG1 - Personal services | 62.667 | 56.905 | 68.429 | 525 |
|  |  | CG2 - Non-personal services | 59.532 | 57.542 | 61.521 | 5255 |
|  | **Quantiles 1 and 2** | Domestic workers | 63.130 | 58.085 | 68.174 | 693 |
|  |  | CG1 - Personal services | 73.353 | 64.030 | 82.675 | 178 |
|  |  | CG2 - Non-personal services | 60.198 | 53.638 | 66.759 | 429 |
| **Age group** | **14 - 34 years old** | Domestic workers | 55.931 | 50.282 | 61.580 | 540 |
|  |  | CG1 - Personal services | 59.321 | 51.375 | 67.268 | 252 |
|  |  | CG2 - Non-personal services | 56.781 | 53.660 | 59.903 | 1966 |
|  | **35 - 49 years old** | Domestic workers | 67.670 | 62.929 | 72.412 | 755 |
|  |  | CG1 - Personal services | 63.258 | 56.178 | 70.338 | 344 |
|  |  | CG2 - Non-personal services | 62.739 | 60.014 | 65.464 | 2637 |
|  | **50 or more years old** | Domestic workers | 71.184 | 66.625 | 75.744 | 691 |
|  |  | CG1 - Personal services | 69.336 | 62.577 | 76.095 | 314 |
|  |  | CG2 - Non-personal services | 59.534 | 56.319 | 62.749 | 2128 |
| **Labor rights met** | **2 or less out of 5** | Domestic workers | 65.668 | 62.669 | 68.666 | 1881 |
|  |  | CG1 - Personal services | 64.752 | 59.298 | 70.206 | 621 |
|  |  | CG2 - Non-personal services | 55.009 | 49.031 | 60.987 | 537 |
|  | **3 or more out of 5** | Domestic workers | 63.030 | 50.175 | 75.885 | 105 |
|  |  | CG1 - Personal services | 61.282 | 53.895 | 68.670 | 289 |
|  |  | CG2 - Non-personal services | 60.254 | 58.325 | 62.184 | 6194 |
| **Chronic condition status** | **No** | Domestic workers | 52.419 | 48.063 | 56.775 | 928 |
|  |  | CG1 - Personal services | 53.286 | 46.601 | 59.972 | 415 |
|  |  | CG2 - Non-personal services | 48.658 | 46.050 | 51.266 | 2983 |
|  | **Yes** | Domestic workers | 77.828 | 74.461 | 81.194 | 1058 |
|  |  | CG1 - Personal services | 73.953 | 68.575 | 79.331 | 495 |
|  |  | CG2 - Non-personal services | 69.697 | 67.445 | 71.949 | 3748 |
| **Household head status** | **No** | Domestic workers | 62.390 | 58.843 | 65.937 | 1397 |
|  |  | CG1 - Personal services | 63.106 | 57.886 | 68.325 | 602 |
|  |  | CG2 - Non-personal services | 57.979 | 55.916 | 60.042 | 5019 |
|  | **Yes** | Domestic workers | 73.642 | 68.560 | 78.724 | 589 |
|  |  | CG1 - Personal services | 63.748 | 56.112 | 71.385 | 308 |
|  |  | CG2 - Non-personal services | 65.883 | 62.427 | 69.338 | 1712 |

Data source: ENAHO 2018 – 2023.

**6.2 Period: Pandemic (Illness symptoms)**

| **Variable** | **Categories** | **Group** | **Pandemic** | | | |
| --- | --- | --- | --- | --- | --- | --- |
|  |  |  | **Point estimate** | **Lower CI 95%** | **Upper CI 95%** | **OBS** |
| **Geographic domain** | **Lima city (capital)** | Domestic workers | 46.825 | 41.722 | 51.928 | 472 |
|  |  | CG1 - Personal services | 37.859 | 26.891 | 48.826 | 122 |
|  |  | CG2 - Non-personal services | 37.936 | 34.668 | 41.204 | 1341 |
|  | **Other cities on the Coast, Highlands and Amazon** | Domestic workers | 58.826 | 55.204 | 62.447 | 1265 |
|  |  | CG1 - Personal services | 62.594 | 55.918 | 69.269 | 478 |
|  |  | CG2 - Non-personal services | 56.082 | 53.700 | 58.463 | 4459 |
| **Education level** | **Incomplete secondary, primary or less** | Domestic workers | 57.894 | 52.993 | 62.795 | 795 |
|  |  | CG1 - Personal services | 54.226 | 41.563 | 66.888 | 136 |
|  |  | CG2 - Non-personal services | 54.631 | 41.553 | 67.709 | 120 |
|  | **Complete secondary or higher education** | Domestic workers | 48.127 | 43.711 | 52.543 | 941 |
|  |  | CG1 - Personal services | 48.988 | 41.351 | 56.626 | 464 |
|  |  | CG2 - Non-personal services | 46.130 | 43.991 | 48.268 | 5680 |
| **Self-reported ethnic group** | **Mixed, white or others** | Domestic workers | 49.289 | 45.498 | 53.080 | 1244 |
|  |  | CG1 - Personal services | 49.025 | 41.664 | 56.386 | 458 |
|  |  | CG2 - Non-personal services | 44.518 | 42.146 | 46.891 | 4667 |
|  | **Indigenous or afrodescendant** | Domestic workers | 60.030 | 53.945 | 66.114 | 493 |
|  |  | CG1 - Personal services | 52.728 | 38.965 | 66.491 | 142 |
|  |  | CG2 - Non-personal services | 53.820 | 49.264 | 58.377 | 1133 |
| **Hourly wage average by quintile** | **Quantiles 4 and 5** | Domestic workers | 45.956 | 41.183 | 50.728 | 756 |
|  |  | CG1 - Personal services | 48.653 | 40.485 | 56.822 | 358 |
|  |  | CG2 - Non-personal services | 45.993 | 43.569 | 48.416 | 4646 |
|  | **Quantiles 1 and 2** | Domestic workers | 65.573 | 59.445 | 71.702 | 460 |
|  |  | CG1 - Personal services | 51.837 | 37.422 | 66.253 | 115 |
|  |  | CG2 - Non-personal services | 44.186 | 35.545 | 52.827 | 328 |
| **Age group** | **14 - 34 years old** | Domestic workers | 52.276 | 46.194 | 58.358 | 530 |
|  |  | CG1 - Personal services | 42.467 | 30.643 | 54.290 | 151 |
|  |  | CG2 - Non-personal services | 41.872 | 38.150 | 45.593 | 1608 |
|  | **35 - 49 years old** | Domestic workers | 53.214 | 48.148 | 58.279 | 597 |
|  |  | CG1 - Personal services | 55.352 | 46.496 | 64.209 | 276 |
|  |  | CG2 - Non-personal services | 48.350 | 45.000 | 51.699 | 2271 |
|  | **50 or more years old** | Domestic workers | 51.064 | 45.130 | 56.998 | 610 |
|  |  | CG1 - Personal services | 52.832 | 40.124 | 65.540 | 173 |
|  |  | CG2 - Non-personal services | 50.072 | 46.470 | 53.674 | 1921 |
| **Labor rights met** | **2 or less out of 5** | Domestic workers | 52.554 | 49.252 | 55.855 | 1649 |
|  |  | CG1 - Personal services | 58.555 | 51.231 | 65.878 | 420 |
|  |  | CG2 - Non-personal services | 41.027 | 33.925 | 48.129 | 409 |
|  | **3 or more out of 5** | Domestic workers | 48.225 | 34.381 | 62.070 | 88 |
|  |  | CG1 - Personal services | 34.918 | 24.039 | 45.798 | 180 |
|  |  | CG2 - Non-personal services | 46.843 | 44.691 | 48.996 | 5391 |
| **Chronic condition status** | **No** | Domestic workers | 40.371 | 35.737 | 45.004 | 767 |
|  |  | CG1 - Personal services | 39.197 | 30.031 | 48.363 | 255 |
|  |  | CG2 - Non-personal services | 33.791 | 30.692 | 36.889 | 2344 |
|  | **Yes** | Domestic workers | 62.738 | 58.655 | 66.821 | 970 |
|  |  | CG1 - Personal services | 60.515 | 51.789 | 69.242 | 345 |
|  |  | CG2 - Non-personal services | 55.889 | 53.219 | 58.559 | 3456 |
| **Household head status** | **No** | Domestic workers | 46.898 | 42.713 | 51.082 | 1095 |
|  |  | CG1 - Personal services | 49.271 | 40.589 | 57.953 | 359 |
|  |  | CG2 - Non-personal services | 44.608 | 42.067 | 47.149 | 3938 |
|  | **Yes** | Domestic workers | 61.096 | 55.887 | 66.306 | 642 |
|  |  | CG1 - Personal services | 51.202 | 41.425 | 60.979 | 241 |
|  |  | CG2 - Non-personal services | 50.758 | 47.117 | 54.398 | 1862 |

Data source: ENAHO 2018 – 2023.

**6.3 Period: Post-pandemic (Illness symptoms)**

| **Variable** | **Categories** | **Group** | **Post-pandemic** | | | |
| --- | --- | --- | --- | --- | --- | --- |
|  |  |  | **Point estimate** | **Lower CI 95%** | **Upper CI 95%** | **OBS** |
| **Geographic domain** | **Lima city (capital)** | Domestic workers | 52.912 | 45.708 | 60.115 | 275 |
|  |  | CG1 - Personal services | 41.148 | 29.154 | 53.143 | 76 |
|  |  | CG2 - Non-personal services | 42.906 | 38.718 | 47.095 | 796 |
|  | **Other cities on the Coast, Highlands and Amazon** | Domestic workers | 61.227 | 56.652 | 65.802 | 694 |
|  |  | CG1 - Personal services | 64.486 | 56.799 | 72.173 | 267 |
|  |  | CG2 - Non-personal services | 62.048 | 59.251 | 64.845 | 2587 |
| **Education level** | **Incomplete secondary, primary or less** | Domestic workers | 63.938 | 57.737 | 70.139 | 417 |
|  |  | CG1 - Personal services | 52.850 | 35.061 | 70.638 | 72 |
|  |  | CG2 - Non-personal services | 55.960 | 39.051 | 72.868 | 76 |
|  | **Complete secondary or higher education** | Domestic workers | 51.090 | 45.118 | 57.063 | 551 |
|  |  | CG1 - Personal services | 51.728 | 43.257 | 60.199 | 271 |
|  |  | CG2 - Non-personal services | 51.816 | 49.161 | 54.471 | 3306 |
| **Self-reported ethnic group** | **Mixed, white or others** | Domestic workers | 54.790 | 49.670 | 59.910 | 698 |
|  |  | CG1 - Personal services | 46.808 | 37.678 | 55.937 | 265 |
|  |  | CG2 - Non-personal services | 48.952 | 46.125 | 51.778 | 2742 |
|  | **Indigenous or afrodescendant** | Domestic workers | 60.866 | 52.586 | 69.146 | 271 |
|  |  | CG1 - Personal services | 67.018 | 51.749 | 82.287 | 78 |
|  |  | CG2 - Non-personal services | 66.527 | 60.857 | 72.197 | 641 |
| **Hourly wage average by quintile** | **Quantiles 4 and 5** | Domestic workers | 54.672 | 48.838 | 60.506 | 545 |
|  |  | CG1 - Personal services | 52.725 | 43.404 | 62.045 | 230 |
|  |  | CG2 - Non-personal services | 51.155 | 48.367 | 53.943 | 2793 |
|  | **Quantiles 1 and 2** | Domestic workers | 69.038 | 59.957 | 78.120 | 174 |
|  |  | CG1 - Personal services | 58.163 | 35.704 | 80.621 | 41 |
|  |  | CG2 - Non-personal services | 45.467 | 33.641 | 57.294 | 149 |
| **Age group** | **14 - 34 years old** | Domestic workers | 49.447 | 40.651 | 58.243 | 263 |
|  |  | CG1 - Personal services | 41.829 | 28.617 | 55.042 | 104 |
|  |  | CG2 - Non-personal services | 47.871 | 43.050 | 52.692 | 939 |
|  | **35 - 49 years old** | Domestic workers | 53.101 | 46.320 | 59.882 | 345 |
|  |  | CG1 - Personal services | 55.613 | 43.091 | 68.135 | 140 |
|  |  | CG2 - Non-personal services | 54.232 | 50.258 | 58.205 | 1312 |
|  | **50 or more years old** | Domestic workers | 65.865 | 59.177 | 72.553 | 361 |
|  |  | CG1 - Personal services | 64.473 | 49.003 | 79.942 | 99 |
|  |  | CG2 - Non-personal services | 55.176 | 50.896 | 59.456 | 1132 |
| **Labor rights met** | **2 or less out of 5** | Domestic workers | 55.705 | 51.063 | 60.347 | 932 |
|  |  | CG1 - Personal services | 56.389 | 46.313 | 66.465 | 223 |
|  |  | CG2 - Non-personal services | 46.027 | 36.437 | 55.617 | 290 |
|  | **3 or more out of 5** | Domestic workers | 70.055 | 51.164 | 88.947 | 37 |
|  |  | CG1 - Personal services | 45.722 | 33.750 | 57.695 | 120 |
|  |  | CG2 - Non-personal services | 52.659 | 49.937 | 55.382 | 3093 |
| **Chronic condition status** | **No** | Domestic workers | 39.524 | 33.155 | 45.893 | 387 |
|  |  | CG1 - Personal services | 36.056 | 23.816 | 48.296 | 133 |
|  |  | CG2 - Non-personal services | 37.519 | 33.569 | 41.470 | 1346 |
|  | **Yes** | Domestic workers | 68.733 | 63.363 | 74.103 | 582 |
|  |  | CG1 - Personal services | 62.674 | 52.794 | 72.554 | 210 |
|  |  | CG2 - Non-personal services | 62.882 | 59.660 | 66.104 | 2037 |
| **Household head status** | **No** | Domestic workers | 53.219 | 47.488 | 58.951 | 580 |
|  |  | CG1 - Personal services | 49.394 | 39.304 | 59.484 | 202 |
|  |  | CG2 - Non-personal services | 47.999 | 44.736 | 51.262 | 2273 |
|  | **Yes** | Domestic workers | 61.330 | 54.195 | 68.464 | 389 |
|  |  | CG1 - Personal services | 55.919 | 43.266 | 68.573 | 141 |
|  |  | CG2 - Non-personal services | 61.512 | 57.201 | 65.823 | 1110 |

Data source: ENAHO 2018 – 2023.

**SUPPLEMENTARY FILE 7**

**Health-Seeking Behaviour outcome values by individual characteristics of domestic workers and comparison groups by period (on percentage scale)**

**7.1 Period: Pre-pandemic (Healthcare-seeking behaviour)**

| **Variable** | **Categories** | **Group** | **Pre-pandemic** | | | |
| --- | --- | --- | --- | --- | --- | --- |
|  |  |  | **Point estimate** | **Lower CI 95%** | **Upper CI 95%** | **OBS** |
| **Geographic domain** | **Lima city (capital)** | Domestic workers | 33.490 | 27.560 | 39.419 | 353 |
|  |  | CG1 - Personal services | 34.994 | 24.731 | 45.256 | 113 |
|  |  | CG2 - Non-personal services | 43.931 | 40.242 | 47.620 | 882 |
|  | **Other cities on the Coast, Highlands and Amazon** | Domestic workers | 26.619 | 23.118 | 30.121 | 943 |
|  |  | CG1 - Personal services | 35.304 | 29.268 | 41.341 | 448 |
|  |  | CG2 - Non-personal services | 39.576 | 37.396 | 41.755 | 3259 |
| **Education level** | **Secondary incomplete, primary or less** | Domestic workers | 33.799 | 28.516 | 39.083 | 634 |
|  |  | CG1 - Personal services | 30.825 | 18.384 | 43.266 | 121 |
|  |  | CG2 - Non-personal services | 35.174 | 22.538 | 47.809 | 109 |
|  | **Secondary complete or higher education** | Domestic workers | 27.722 | 23.040 | 32.405 | 662 |
|  |  | CG1 - Personal services | 36.070 | 29.477 | 42.662 | 440 |
|  |  | CG2 - Non-personal services | 41.919 | 39.773 | 44.066 | 4031 |
| **Self-reported ethnic group** | **Mixed, white or others** | Domestic workers | 29.707 | 25.681 | 33.733 | 956 |
|  |  | CG1 - Personal services | 37.096 | 30.188 | 44.004 | 412 |
|  |  | CG2 - Non-personal services | 41.602 | 39.137 | 44.067 | 3169 |
|  | **Indigenous or afrodescendant** | Domestic workers | 32.482 | 25.303 | 39.661 | 340 |
|  |  | CG1 - Personal services | 28.439 | 17.422 | 39.455 | 149 |
|  |  | CG2 - Non-personal services | 42.010 | 37.710 | 46.309 | 972 |
| **Hourly wage average by quintile** | **Quantiles 4 and 5** | Domestic workers | 30.773 | 25.077 | 36.469 | 406 |
|  |  | CG1 - Personal services | 39.532 | 31.379 | 47.684 | 316 |
|  |  | CG2 - Non-personal services | 43.875 | 41.470 | 46.281 | 3212 |
|  | **Quantiles 1 and 2** | Domestic workers | 30.840 | 25.216 | 36.465 | 458 |
|  |  | CG1 - Personal services | 27.927 | 16.772 | 39.082 | 121 |
|  |  | CG2 - Non-personal services | 31.749 | 24.329 | 39.170 | 268 |
| **Age group** | **14 - 34 years old** | Domestic workers | 19.103 | 13.515 | 24.691 | 305 |
|  |  | CG1 - Personal services | 35.662 | 24.770 | 46.554 | 154 |
|  |  | CG2 - Non-personal services | 34.697 | 30.948 | 38.445 | 1134 |
|  | **35 - 49 years old** | Domestic workers | 30.944 | 25.485 | 36.403 | 507 |
|  |  | CG1 - Personal services | 35.812 | 26.019 | 45.605 | 203 |
|  |  | CG2 - Non-personal services | 41.863 | 38.604 | 45.123 | 1641 |
|  | **50 or more years old** | Domestic workers | 37.791 | 31.836 | 43.745 | 484 |
|  |  | CG1 - Personal services | 33.561 | 23.545 | 43.577 | 204 |
|  |  | CG2 - Non-personal services | 50.806 | 46.873 | 54.740 | 1366 |
| **Number of labor rights met (out of 5)** | **2 or less** | Domestic workers | 30.224 | 26.523 | 33.926 | 1232 |
|  |  | CG1 - Personal services | 34.882 | 27.376 | 42.387 | 385 |
|  |  | CG2 - Non-personal services | 28.519 | 22.126 | 34.912 | 309 |
|  | **3 or more** | Domestic workers | 32.720 | 18.370 | 47.070 | 64 |
|  |  | CG1 - Personal services | 35.540 | 25.462 | 45.618 | 176 |
|  |  | CG2 - Non-personal services | 42.915 | 40.682 | 45.148 | 3832 |
| **Chronic condition status** | **No** | Domestic workers | 26.550 | 21.140 | 31.960 | 479 |
|  |  | CG1 - Personal services | 29.716 | 20.450 | 38.982 | 212 |
|  |  | CG2 - Non-personal services | 33.630 | 30.365 | 36.895 | 1471 |
|  | **Yes** | Domestic workers | 32.871 | 28.674 | 37.068 | 817 |
|  |  | CG1 - Personal services | 39.330 | 32.105 | 46.555 | 349 |
|  |  | CG2 - Non-personal services | 46.723 | 43.940 | 49.505 | 2670 |
| **Household head status** | **No** | Domestic workers | 29.057 | 24.878 | 33.236 | 865 |
|  |  | CG1 - Personal services | 35.758 | 28.456 | 43.061 | 369 |
|  |  | CG2 - Non-personal services | 39.278 | 36.826 | 41.729 | 3002 |
|  | **Yes** | Domestic workers | 33.452 | 27.355 | 39.549 | 431 |
|  |  | CG1 - Personal services | 33.590 | 23.539 | 43.640 | 192 |
|  |  | CG2 - Non-personal services | 48.946 | 44.786 | 53.106 | 1139 |

Data source: ENAHO 2018 – 2023.

**7.2 Period: Pandemic (Healthcare-seeking behaviour)**

| **Variable** | **Categories** | **Group** | **Pandemic** | | | |
| --- | --- | --- | --- | --- | --- | --- |
|  |  |  | **Point estimate** | **Lower CI 95%** | **Upper CI 95%** | **OBS** |
| **Geographic domain** | **Lima city (capital)** | Domestic workers | 22.481 | 16.239 | 28.724 | 215 |
|  |  | CG1 - Personal services | 28.481 | 13.895 | 43.068 | 47 |
|  |  | CG2 - Non-personal services | 40.490 | 35.204 | 45.776 | 467 |
|  | **Other cities on the Coast, Highlands and Amazon** | Domestic workers | 19.905 | 16.125 | 23.686 | 766 |
|  |  | CG1 - Personal services | 26.466 | 18.892 | 34.040 | 279 |
|  |  | CG2 - Non-personal services | 35.465 | 32.634 | 38.296 | 2,466 |
| **Education level** | **Secondary incomplete, primary or less** | Domestic workers | 27.634 | 21.720 | 33.548 | 489 |
|  |  | CG1 - Personal services | 25.945 | 9.191 | 42.699 | 85 |
|  |  | CG2 - Non-personal services | 39.355 | 24.252 | 54.458 | 70 |
|  | **Secondary complete or higher education** | Domestic workers | 15.272 | 11.183 | 19.361 | 492 |
|  |  | CG1 - Personal services | 27.577 | 19.516 | 35.638 | 241 |
|  |  | CG2 - Non-personal services | 37.622 | 34.758 | 40.485 | 2,863 |
| **Self-reported ethnic group** | **Mixed, white or others** | Domestic workers | 18.992 | 14.986 | 22.998 | 673 |
|  |  | CG1 - Personal services | 28.167 | 19.577 | 36.756 | 242 |
|  |  | CG2 - Non-personal services | 37.512 | 34.392 | 40.632 | 2,258 |
|  | **Indigenous or afrodescendant** | Domestic workers | 25.853 | 18.693 | 33.013 | 308 |
|  |  | CG1 - Personal services | 24.745 | 11.760 | 37.730 | 84 |
|  |  | CG2 - Non-personal services | 38.218 | 31.578 | 44.858 | 675 |
| **Hourly wage average by quintile** | **Quantiles 4 and 5** | Domestic workers | 23.993 | 17.884 | 30.102 | 384 |
|  |  | CG1 - Personal services | 24.193 | 15.686 | 32.700 | 197 |
|  |  | CG2 - Non-personal services | 38.169 | 35.016 | 41.322 | 2,330 |
|  | **Quantiles 1 and 2** | Domestic workers | 18.851 | 12.171 | 25.531 | 308 |
|  |  | CG1 - Personal services | 23.371 | 7.281 | 39.461 | 59 |
|  |  | CG2 - Non-personal services | 29.058 | 17.895 | 40.221 | 161 |
| **Age group** | **14 - 34 years old** | Domestic workers | 17.942 | 11.551 | 24.333 | 291 |
|  |  | CG1 - Personal services | 21.455 | 6.229 | 36.680 | 72 |
|  |  | CG2 - Non-personal services | 32.714 | 27.535 | 37.893 | 728 |
|  | **35 - 49 years old** | Domestic workers | 22.672 | 16.653 | 28.691 | 340 |
|  |  | CG1 - Personal services | 26.816 | 16.363 | 37.269 | 155 |
|  |  | CG2 - Non-personal services | 37.502 | 33.392 | 41.612 | 1,157 |
|  | **50 or more years old** | Domestic workers | 22.511 | 16.559 | 28.463 | 350 |
|  |  | CG1 - Personal services | 36.346 | 20.736 | 51.955 | 99 |
|  |  | CG2 - Non-personal services | 44.128 | 39.149 | 49.107 | 1,048 |
| **Number of labor rights met (out of 5)** | **2 or less** | Domestic workers | 20.848 | 17.171 | 24.526 | 942 |
|  |  | CG1 - Personal services | 26.966 | 18.527 | 35.406 | 246 |
|  |  | CG2 - Non-personal services | 28.833 | 18.151 | 39.516 | 173 |
|  | **3 or more** | Domestic workers | 25.896 | 7.194 | 44.598 | 39 |
|  |  | CG1 - Personal services | 28.066 | 10.457 | 45.675 | 80 |
|  |  | CG2 - Non-personal services | 38.402 | 35.502 | 41.302 | 2,760 |
| **Chronic condition status** | **No** | Domestic workers | 16.238 | 10.781 | 21.695 | 341 |
|  |  | CG1 - Personal services | 16.557 | 7.857 | 25.258 | 111 |
|  |  | CG2 - Non-personal services | 32.170 | 27.110 | 37.230 | 831 |
|  | **Yes** | Domestic workers | 23.968 | 19.219 | 28.716 | 640 |
|  |  | CG1 - Personal services | 34.022 | 23.446 | 44.597 | 215 |
|  |  | CG2 - Non-personal services | 40.203 | 36.714 | 43.692 | 2,102 |
| **Household head status** | **No** | Domestic workers | 17.516 | 13.158 | 21.874 | 572 |
|  |  | CG1 - Personal services | 33.041 | 23.008 | 43.074 | 196 |
|  |  | CG2 - Non-personal services | 34.901 | 31.536 | 38.266 | 1,905 |
|  | **Yes** | Domestic workers | 25.796 | 20.034 | 31.558 | 409 |
|  |  | CG1 - Personal services | 17.417 | 8.966 | 25.868 | 130 |
|  |  | CG2 - Non-personal services | 43.873 | 39.150 | 48.596 | 1,028 |

Data source: ENAHO 2018 – 2023.

**7.3 Period: Post-pandemic (Healthcare-seeking behaviour)**

| **Variable** | **Categories** | **Group** | **Post-pandemic** | | | |
| --- | --- | --- | --- | --- | --- | --- |
|  |  |  | **Point estimate** | **Lower CI 95%** | **Upper CI 95%** | **OBS** |
| **Geographic domain** | **Lima city (capital)** | Domestic workers | 35.014 | 25.579 | 44.449 | 140 |
|  |  | CG1 - Personal services | 24.722 | 8.534 | 40.910 | 33 |
|  |  | CG2 - Non-personal services | 43.482 | 37.162 | 49.803 | 336 |
|  | **Other cities on the Coast, Highlands and Amazon** | Domestic workers | 25.981 | 20.952 | 31.010 | 423 |
|  |  | CG1 - Personal services | 32.525 | 22.032 | 43.018 | 167 |
|  |  | CG2 - Non-personal services | 37.879 | 34.541 | 41.218 | 1,575 |
| **Education level** | **Secondary incomplete, primary or less** | Domestic workers | 33.589 | 25.338 | 41.841 | 266 |
|  |  | CG1 - Personal services | 38.383 | 14.440 | 62.327 | 42 |
|  |  | CG2 - Non-personal services | 61.688 | 43.092 | 80.284 | 49 |
|  | **Secondary complete or higher education** | Domestic workers | 28.698 | 21.158 | 36.238 | 296 |
|  |  | CG1 - Personal services | 26.893 | 17.355 | 36.432 | 158 |
|  |  | CG2 - Non-personal services | 39.668 | 36.275 | 43.060 | 1,861 |
| **Self-reported ethnic group** | **Mixed, white or others** | Domestic workers | 29.476 | 22.721 | 36.232 | 388 |
|  |  | CG1 - Personal services | 31.169 | 20.642 | 41.697 | 145 |
|  |  | CG2 - Non-personal services | 40.255 | 36.394 | 44.117 | 1,469 |
|  | **Indigenous or afrodescendant** | Domestic workers | 33.755 | 24.091 | 43.419 | 175 |
|  |  | CG1 - Personal services | 25.184 | 7.664 | 42.704 | 55 |
|  |  | CG2 - Non-personal services | 40.557 | 33.258 | 47.856 | 442 |
| **Hourly wage average by quintile** | **Quantiles 4 and 5** | Domestic workers | 31.698 | 24.133 | 39.263 | 302 |
|  |  | CG1 - Personal services | 28.317 | 16.823 | 39.811 | 136 |
|  |  | CG2 - Non-personal services | 39.309 | 35.497 | 43.120 | 1,564 |
|  | **Quantiles 1 and 2** | Domestic workers | 31.045 | 19.447 | 42.642 | 117 |
|  |  | CG1 - Personal services | 31.592 | 4.350 | 58.835 | 23 |
|  |  | CG2 - Non-personal services | 49.336 | 32.178 | 66.493 | 73 |
| **Age group** | **14 - 34 years old** | Domestic workers | 22.791 | 11.775 | 33.806 | 139 |
|  |  | CG1 - Personal services | 26.931 | 12.234 | 41.629 | 54 |
|  |  | CG2 - Non-personal services | 40.319 | 33.871 | 46.766 | 470 |
|  | **35 - 49 years old** | Domestic workers | 32.754 | 23.644 | 41.864 | 190 |
|  |  | CG1 - Personal services | 31.235 | 14.876 | 47.594 | 81 |
|  |  | CG2 - Non-personal services | 39.736 | 34.510 | 44.963 | 743 |
|  | **50 or more years old** | Domestic workers | 34.277 | 25.677 | 42.878 | 234 |
|  |  | CG1 - Personal services | 29.180 | 13.600 | 44.760 | 65 |
|  |  | CG2 - Non-personal services | 41.148 | 35.572 | 46.725 | 698 |
| **Number of labor rights met (out of 5)** | **2 or less** | Domestic workers | 29.886 | 24.280 | 35.492 | 538 |
|  |  | CG1 - Personal services | 25.645 | 14.994 | 36.297 | 135 |
|  |  | CG2 - Non-personal services | 42.548 | 28.735 | 56.361 | 146 |
|  | **3 or more** | Domestic workers | 42.764 | 16.034 | 69.495 | 25 |
|  |  | CG1 - Personal services | 35.362 | 18.880 | 51.845 | 65 |
|  |  | CG2 - Non-personal services | 40.087 | 36.668 | 43.506 | 1,765 |
| **Chronic condition status** | **No** | Domestic workers | 15.602 | 8.700 | 22.504 | 171 |
|  |  | CG1 - Personal services | 20.139 | 7.488 | 32.789 | 59 |
|  |  | CG2 - Non-personal services | 36.429 | 30.250 | 42.609 | 559 |
|  | **Yes** | Domestic workers | 37.062 | 29.882 | 44.241 | 392 |
|  |  | CG1 - Personal services | 32.724 | 21.251 | 44.197 | 141 |
|  |  | CG2 - Non-personal services | 42.081 | 37.947 | 46.214 | 1,352 |
| **Household head status** | **No** | Domestic workers | 28.354 | 21.008 | 35.699 | 317 |
|  |  | CG1 - Personal services | 24.819 | 13.487 | 36.152 | 107 |
|  |  | CG2 - Non-personal services | 40.619 | 36.525 | 44.713 | 1,191 |
|  | **Yes** | Domestic workers | 33.866 | 25.255 | 42.477 | 246 |
|  |  | CG1 - Personal services | 35.222 | 20.750 | 49.694 | 93 |
|  |  | CG2 - Non-personal services | 39.758 | 33.973 | 45.544 | 720 |

Data source: ENAHO 2018 – 2023.
